# Guided monocyte fate to FRβ/CD163^+^ S1 macrophage antagonises atopic dermatitis via fibroblastic matrices in mouse hypodermis

**DOI:** 10.1007/s00018-024-05543-2

**Published:** 2024-12-25

**Authors:** Yu-Tung Li, Eiichi Takaki, Yuya Ouchi, Katsuto Tamai

**Affiliations:** 1 Department of Stem Cell Therapy Science, Graduate School of Medicine, Osaka University, Suita, Osaka 565-0871 Japan; 2 StemRIM Inc., Ibaraki, Osaka 567-0085 Japan

**Keywords:** Cell identity, Heterogeneity, Efferocytosis, Niche, Pan-tissue, Extracellular matrix

## Abstract

**Abstract:**

Macrophages are versatile myeloid leukocytes with flexible cellular states to perform diverse tissue functions beyond immunity. This plasticity is however often hijacked by diseases to promote pathology. Scanning kinetics of macrophage states by single-cell transcriptomics and flow cytometry, we observed atopic dermatitis drastically exhausted a resident subtype S1. Characterized by FRβ/CD163 expression, S1 exhibited strong efferocytosis and chemoattracted monocytes and eosinophils. Here we have delineated mechanisms regulating monocyte decision to acquire S1 identity in skin. During M-CSF driven macrophage differentiation in healthy skin, FRβ was expressed via intrinsic control of STAT6 and ALK5 activities, and did not require heterotypic cellular crosstalk. In contrast, CD163 expression required exposure to fibroblastic secretion. This process depended on SHP1 activity and involved STAT5 inactivation. Suppressed STAT5 activity caused CD163 expression and rendered macrophage insensitive to further induction by fibroblasts. Parsing coculture experiments with in silico ligand expression, we identified laminin-α2 and type-V collagen secreted by hypodermal fibroblasts as CD163-driving factors. S1 identity loss in AD followed a stepwise cascade: reduced laminins availability first dampened CD163 expression, IL4 and TGFβ subsequently acted on CD163^lo/−^ cells to downregulate FRβ. In AD skin, we showed that imitating this fibroblast-macrophage crosstalk with exogenous laminin-211 encouraged monocyte differentiation to S1 macrophages, fostered homeostatic commitment of extravasated eosinophils, and alleviated dermatitis. Hence, we demonstrated that reinforcing a steady-state cue from hypodermal fibroblasts could override maladaptive pressure on macrophage and restored tissue homeostasis.

**Graphical Abstract:**

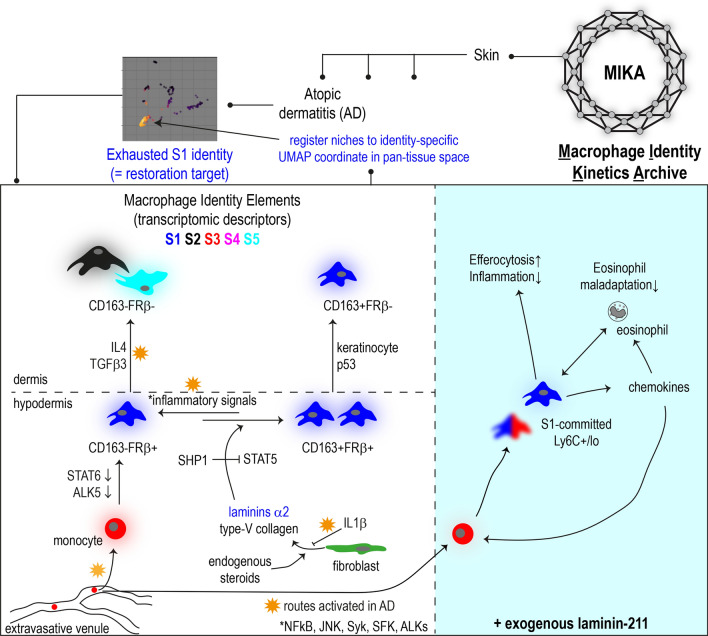

**Supplementary Information:**

The online version contains supplementary material available at 10.1007/s00018-024-05543-2.

## Introduction

Macrophages have long been considered as inflammatory leukocytes since their first recognition in the nineteenth century. Only until recently, genomics began to reveal their charisma in modulating various tissue functions. Such functional multitude is enable with macrophage constantly adapting its transcriptome to its surroundings [1, 2]. While macrophage functions could be influenced by lineage origins [3–5], in situ adaptation often has dominant impacts [2, 6]. The interplay between cellular plasticity and diverse local physiology allows macrophage to serve far-reaching functions of cardiac electrical conductance, neural development, stem cell trafficking and thermogenesis [7–10]. During inflammatory clearance, cellular plasticity permits regulated transition to a resolving phenotype to avoid excessive responses. One trigger for macrophage transition to resolution is a form of phagocytosis, known as efferocytosis, specialised to engulf apoptotic cells that accumulate across the inflammatory course [11]. Efferocytosis is a multi-step process initiated by sensing chemotactic cues from apoptotic cells followed by migration, cell–cell contact establishment, and engulfment. The process leads to cellular responses to release immunomodulatory factors that are pivotal to tame inflammation and maintain homeostasis.

In proceeding or chronic diseases, nevertheless, cellular plasticity has detrimental impacts since macrophages are often shaped into pathological phenotypes. These macrophages propagate tissue pathology either directly or via interaction with other effector cells. Macrophages in arthritis synovium generate bone-resorbing osteoclasts [12]; tumour-associated macrophages guard the lesion by suppressing immune assaults [13]. Various studies have shown that identification of disease-associated macrophages and their elimination are effective to offer therapeutic benefits [6, 14, 15]. Nevertheless, maladaptive pressure remains in the tissues. Alternatively, we focused on the intercellular crosstalk cues fostering physiological development of beneficial macrophages which become under-represented in diseases, and attempted to restore the desirable macrophage populations by amplifying the natural cues. Successful restoration requires identification of the disease-antagonizing macrophage, its housekeeping niches and understanding of how these niches orchestrate its formation at homeostasis.

Atopic dermatitis (AD) is a life quality deteriorating allergic disease with recurring inflammation, pruritis, neo-vascularization and disrupted epidermal barrier functions. While the immunopathology is driven by activation of Th2/Th22, other Th axes may be involved depending on disease subtype, ethnicity, and age. Besides T cell immunity, studies have reported regulatory roles of other cells including myeloid leukocytes [16, 17]. In mice, topical application of a vitamin-D analogue MC903 triggers thymic stromal lymphopoietin (TSLP) secretion from keratinocyte to activate both innate and adaptive immune compartments to initiate AD resembling clinical traits in human [18]. Humoral and cellular immunological landscape in AD skin significantly differs from homeostatic conditions with upregulated cytokines and leukocyte accumulation. How these aberrations affect the detailed cell states of macrophages and how different macrophage states regulate the functions of the newly extravasated leukocytes remain obscure.

Extracellular matrix (ECM) is a group of proteins highly synthesized by fibroblasts. In skin, it provides an attachment surface for cells and maintains tissue structural integrity. It also acts as a scaffold to bind growth factors to control their bioavailability and thus regulate tissue remodelling [19]. Long believed as an inert structure, cumulating evidence suggested ECM may directly regulate cell functions and its composition is kinetically regulated by the tissue environment. In AD, for example, upregulated expression of periostin promoted TSLP secretion and pruritis by binding αvβ3 integrin on keratinocytes and sensory neurons [20, 21]. Laminins constitute a subfamily of ECM that are synthesized as heterotrimers abundantly found in basement membranes throughout the body. Tissue distribution and the binding ligands vary by isoforms and may even depend on developmental stages [22]. Once synthesized and secreted, laminins are organized and structurally bridged with other ECM into basement membrane. In skin, laminin α3, α5, β1, β2, β3, γ1 and γ2 are abundant in epidermal basement membrane, while laminin α4, α5, β1, β2 and γ1 are rich at the vascular basement membrane [23]. Besides providing structural support and constituting the basement membrane barriers, alike other ECM, laminins are bioactive and capable to modulate cell functions. Vascular laminins have been shown to mediate mechano-sensing in endothelial cells, regulate leukocyte extravasation and support macrophage differentiation [24–26]. Whether and how laminins regulate macrophage cell state transition remain unexplored.

In MC903-induced AD, we detected prominent reduction of a resident macrophage subtype S1 marked by FRβ/CD163 expression. We described S1 as a macrophage subtype specialised to maintain tissue homeostasis via efferocytosis and turnover of monocytes and eosinophils via chemokine secretion. Integrating transcriptomics and multi-cell type coculture experiments, we showed keratinocytes and fibroblasts regulated development of S1 macrophage identity in skin. Macrophages needed to be distant from keratinocyte to maintain the intrinsic FRβ expression. Exposure to fibroblastic laminin-α2 induced CD163 expression. We found that hypodermis, where keratinocytes are absent and fibroblastic laminins deposit, harboured macrophage of the strongest S1 identity (FRβ^+^CD163^+^). Limited bioavailability of laminins and monocyte extravasation in AD caused reduced CD163 expression and licensed AD cytokines to suppress S1 identity. Treating S1 identity low AD skin with exogenous laminin-α2 directed monocytes to S1 and provoked homeostatic commitment of eosinophils to alleviate inflammation. Together, our work demonstrated novel involvement of hypodermal fibroblasts to establish S1 macrophage identity that was lost in AD. Mimicking this crosstalk with laminin-α2 could restore macrophage identity balance and alleviate AD pathology.

## Results

### Progressive AD exhausts FRβ/CD163^+^ tissue resident S1 macrophage

**Fig. 1:**
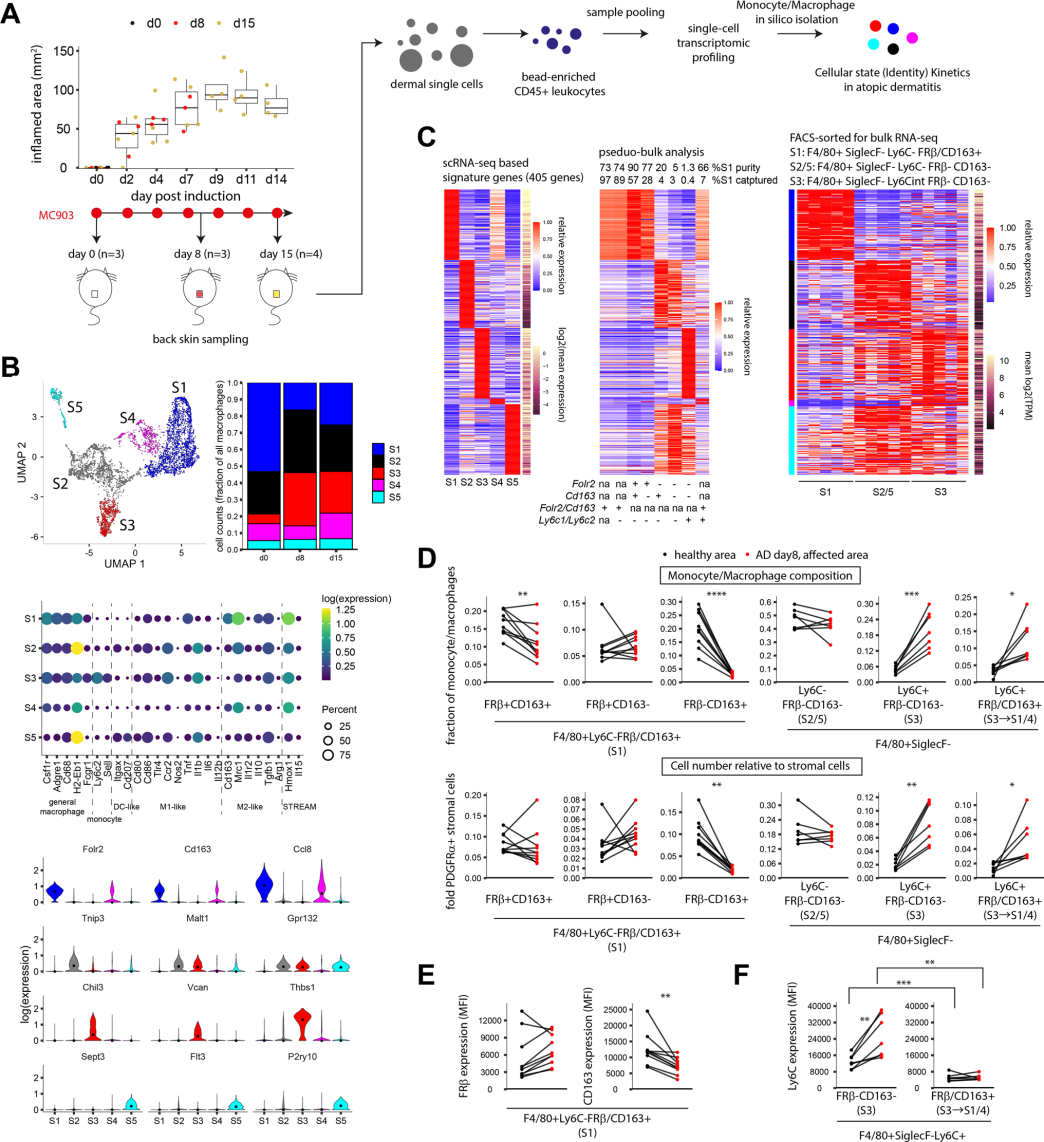
Dermal macrophage subtype S1 is exhausted in atopic dermatitis. **A** Atopic dermatitis (AD) was induced by MC903 on shaved back skin. Skin samples were collected in time course and prepared for single-cell transcriptome analysis. Cells were pooled from indicated number (*n*) of individual mice. **B** Macrophages were isolated from cell clusters on UMAP and examined for subtype dynamics during AD progression. **C** Signature genes were identified for each macrophage subtype. Signature expression of indicated pseudo-bulk sc subsets or bulk transcriptomes of FACS-sorted subtypes was examined. *n* = 5 healthy mice for each subtype bulks individually analysed. **D** On day 8 post AD induction, dermal macrophages were isolated from dermatitis and healthy regions in pairs for analysis of the indicated MOMF subsets. **E**,**F** Expression of FRβ, CD163 and Ly6C on indicated MOMF subsets were examined. *n* = 7–10 mice in **D**–**F**. Groups were compared by paired *t* test or Wilcoxon signed-sum test followed by Bonferroni correction in **D**–**F**. * *p* < 0.05, ** *p* < 0.01, *** *p* < 0.001, **** *p* < 0.0001

To trace the dynamic macrophage states in progressive MC903-induced AD, we isolated leukocyte-enriched cells from dorsal skin in time course and surveyed their transcriptomes by single-cell (sc) RNA-seq (Fig. 1A; Fig. S1A). We identified 5 subsets of monocytes/macrophages (MOMF), with mixed M1/M2 characters and differing from dermal STREAM [27] (*H2-Eb1*^*−*^*Hmox*^+^*Il10*^*−*^*Il15*^+^). We thus hereafter refer them as macrophage subtypes. AD induction caused a substantial decline of subtype 1 (S1) and a surge in S3. S1/4 expresses high levels of *Cd163* and *Folr2*. S2/5 are DC-like (*Itgax*^+^) and express the highest level of MHCII (*H2-Eb1*) with S5 uniquely expressing the Langerhans cell marker *Cd207*. S3 contains *Ly6c2*^+^*Sell*^+^ monocytes, with immediate transcriptomic continuum to S2 (Fig. 1B). To facilitate flow cytometric identification of subtypes, we constructed a gene signature with 405 subtype-specific genes and evaluated if *Ly6c1/2*, *Folr2* and *Cd163* mark subtypes. Signature expression were examined in cell pseudo-bulks aggregated by marker combinations. S1 was best captured as *Ly6c1/2*^*−*^*Folr2/Cd163*^+^ (89%). *Ly6c1/2-Folr2-Cd163*^+*/−*^ showed strong S2/5 characters; *Ly6c1/2*^+^*Folr2*^*−*^*Cd163*^*−*^ solely corresponded to S3. *Folr2/Cd163* expression in *Ly6c1/2*^+^ monocytes correlated to gain of S1/4 characters and suggested S1/4 transition. Signature gene expression of Ly6C^−^CD163/FRβ^+^, Ly6C^−^CD163^−^FRβ^−^ and Ly6C^int^CD163^−^FRβ^−^, isolated from the dermal F4/80^+^SiglecF^−^ MOMF and analysed by bulk RNA-seq, confirmed them as bulk representations of S1, S2/5 and S3 respectively (Fig. 1C).

To verify macrophage subtype kinetics in AD by protein antigens, we compared S1-marking FRβ/CD163 surface expression and macrophage subtype proportion in paired AD/healthy dorsal skin regions of PDGFRα-H2BGFP knock-in mice (Fig. S1B–C). PDGFRα^+^ stromal cells, a less dynamic population in inflammation than leukocytes, in these mice were labelled by GFP allowing evaluation of MOMF cell numbers relative to the stromal population. FRβ/CD163 expression was specific on F4/80^+^SiglecF^−^ MOMF but minimal on SiglecF^+^ eosinophils and stromal cells (Fig. S1D–E). Fading S1 characters were evident as reduced Ly6C^−^FRβ^+^CD163^+^ proportions within macrophages and reduced surface CD163 expression on Ly6C^−^FRβ/CD163^+^ S1. The latter indicates S1 decline was an active process but not passively caused by monocyte influx. FRβ surface expression on remnant S1 was unaffected. Extravasated Ly6C^+^ monocytes contained both FRβ^−^CD163^−^ S3 and FRβ/CD163^+^ S1/4-committed cells. FRβ/CD163^+^ monocytes were differentiating to macrophages as indicated by significantly less Ly6C expression (Fig. 1D–F; Fig. S2). Diminished S1 character in progressive AD and its homeostatic presence suggested S1 as a restorative target.

### Fibroblasts and keratinocytes regulate phenotype of S1 macrophage

**Fig. 2:**
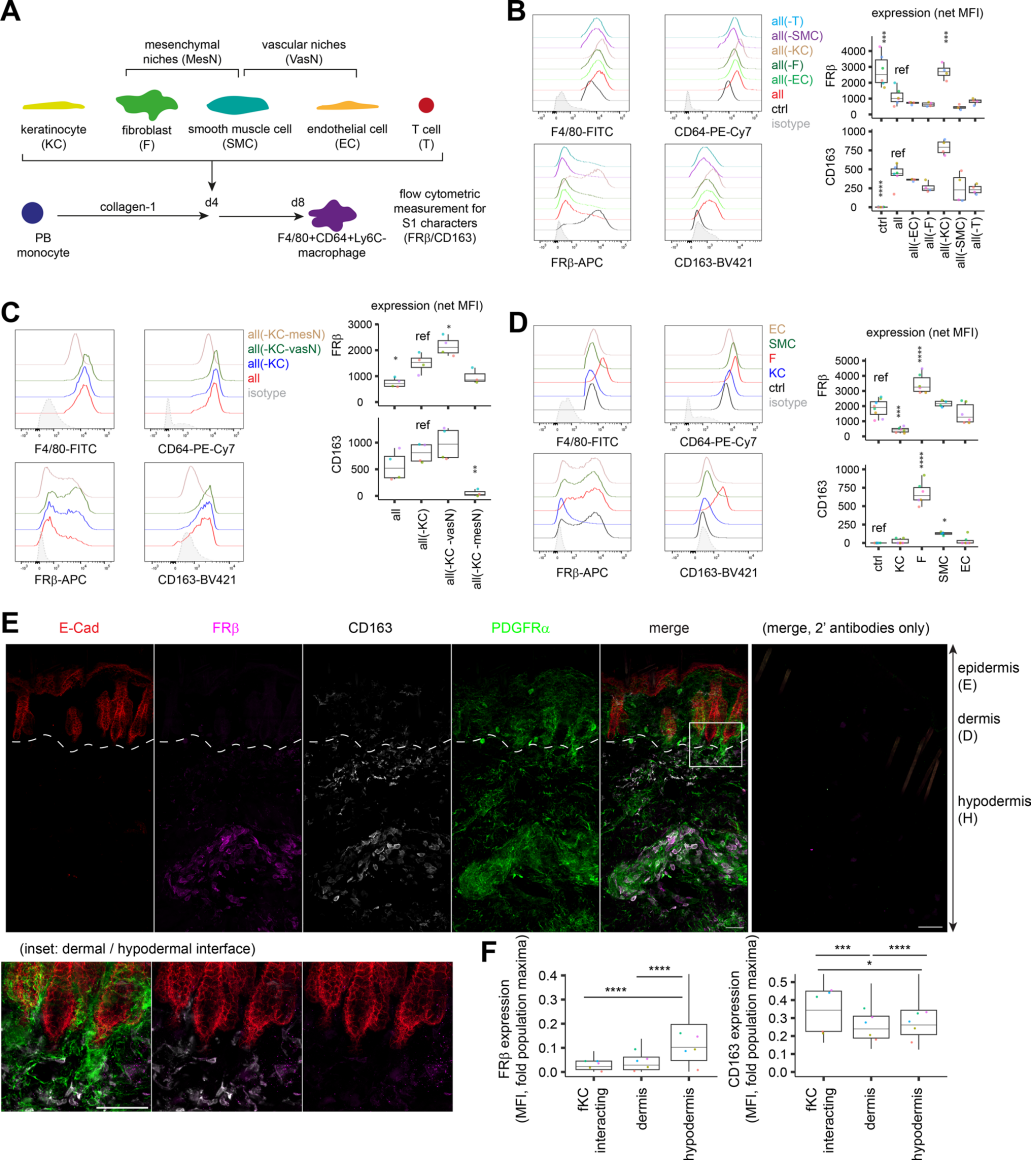
Fibroblasts and keratinocytes modulate macrophage expression of S1-marking FRβ and CD163 in skin. **A** PB leukocytes were differentiated on collagen-I coated surface with 20 ng/ml M-CSF for 4 days. Medium was replaced with coculture medium containing the indicated heterotypic cells and cells were cocultured for another 4 days. (B-D) F4/80^+^CD64^+^Ly6C^−^ macrophage phenotype was analysed by flow cytometry on day 8. *n* = 6 batches for ctrl and complete coculture (all), and *n* = 4 for the rest in **B**. *n* = 3 batches for all-KC-mesN, *n* = 4 batches for the rest in **C**. *n* = 8 batches for ctrl, *n* = 6 batches for cocultures with EC, fibroblasts and KC, *n* = 4 for SMC coculture in **D**. Each batch (colour-coded) is a biological replicate with cells derived from an individual mouse. **E** CD163^+^ S1 macrophages interacting with E-Cad+ follicular keratinocytes (fKC), in dermis and in hypodermis were examined in healthy skin by volumetric confocal microscopy and analysed with IMARIS. **F** Expression of FRβ and CD163 on CD163+ S1 in each position were quantified. Inset shows FRβ^+^CD163^+^ S1 accumulated to hypodermal facet. *n* = 34 fKC-interacting, 434 dermal and 944 hypodermal CD163^+^ cells from 5 mice. E, epidermis; D, dermis; H, hypodermis. Scales, 50 μm. Colour-coded dots indicated median of each mouse. Groups were compared to batch-specific reference sample by 2-way ANOVA followed by Dunnett’s test in **B**–**D** or by Kruskal–Wallis test with Bonferroni correction in **F**. * *p* < 0.05, ** *p* < 0.01, *** *p* < 0.001, **** *p* < 0.0001

To determine how the CD163/FRβ marked S1 identity is formed in healthy skin, we performed coculture assays with differentiating peripheral blood monocytes on collagen-1-coated surface to mimick monocytes extravasating to skin [28] in the presence of M-CSF, a maintenance factor for MOMF primarily secreted by fibroblasts in normal skin (Fig. 2A; Fig. S3A). FRβ, but not CD163, was intrinsically expressed during macrophage differentiation in the absence of heterotypic cellular interference (Fig. S3B). Since coexpression of *Cd163* in *Folr2*^+^ macrophage corresponded to a stronger S1 character in vivo, we examined whether signals to induce CD163 expression are provided by other dermal cell types. In a coculture comprising keratinocytes (KC), fibroblasts, endothelial cells (EC), smooth muscle cells (SMC) and T cells, CD163 expression on macrophage was evident. Of notes, this complete coculture accompanied reduced FRβ expression that was restored by omitting KC, indicating suppressive roles of KC. Omitting individual cell types did not affect CD163 expression (Fig. 2B; Fig. S3C). In KC-absent cocultures, removing the mesenchymal niches (fibroblasts and SMC), but not the vascular niches (EC and SMC), fully suppressed CD163 induction (Fig. 2C). Coculturing monocytes with KC or fibroblasts alone confirmed them respectively as FRβ suppressor and CD163 inducer (Fig. 2D).

Given the distinct localisation of fibroblast and KC in skin, we assessed whether CD163/FRβ expression on the nearby macrophages may be modulated. The native residence of CD163^+^ S1 in healthy skin was analysed with volumetric confocal microscopy. CD163^+^ MOMF were observed to span across both dermis and hypodermis, with those in dermis expressing significantly less FRβ, resulting in a spatial partition of dermal CD163^+^FRβ^−^ and hypodermal CD163^+^FRβ^+^ S1 (Fig. 2E–F; Fig. S4). This localisation pattern is coherent to the distribution of ubiquitous fibroblasts and FRβ-suppressive epithelial cells, including follicular KC that extends from the epidermis into dermal layers. Together, these findings suggested fibroblasts and KC provide extrinsic signals during macrophage differentiation to shape S1 phenotypes.

### Fibroblastic laminins intervene macrophage differentiation to form CD163^+^ S1 identity

**Fig. 3:**
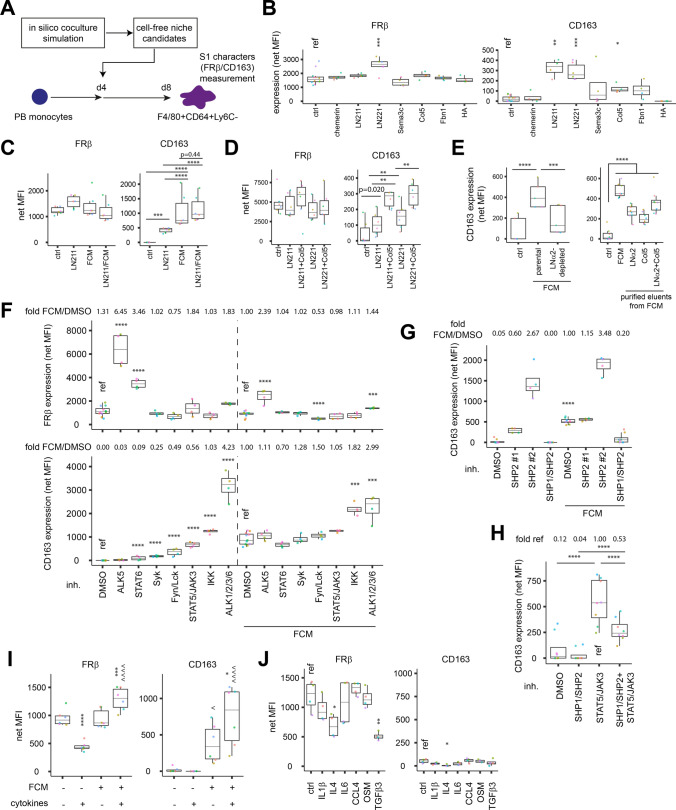
Fibroblast-derived niches supports macrophage CD163 expression via STAT5 inhibition. **A** Coculture experiments described in Fig. 2 were simulated in silico to identify potential FRβ/CD163 modulating cytokines and fibroblast-derived ligands. **B** Ligand candidates were screened for FRβ/CD163 modulation capacity with PB-derived macrophages. *n* = 6 batches for LN221 and 4 batches each for others. **C**,**D** Combinations of the indicated CD163-inducing factors were assessed for modulation strengths with PB-derived macrophages. *n* = 6 batches each for both (**C**, **D**). **E** (Left) FCM was depleted for LNa2 and assessed for CD163 induction capacity. *n* = 5 batches. (Right) Indicated components were affinity purified from FCM and assessed for CD163 induction capacity. *n* = 8 batches. **F**–**H** FRβ/CD163 expression in the presence of inhibitors and/or FCM were measured. *n* = 12 batches for each DMSO ctrl and 4 batches for others in **F**. *n* = 4 batches for SHP2 inhibitors and 8 batches for others in **G**. *n* = 8 batches each in **H**–**J** With or without dermal fibroblasts conditioned medium (FCM, 4-day in coculture medium), FRβ/CD163 modulation capacity of cytokines in a cocktail (**I**) or individually (**J**) were analysed. *n* = 6 batches each in **I** and 4 batches each in **J**. Each batch is a biological replicate with cells derived from an individual mouse. Groups were analysed by 2-way ANOVA and compared to reference sample by Dunnett’s test in **B**, **F**, and **J**, or to other groups by Tukey’s test in **C**, **E**, **H**, and **I**, or by paired *t* test with Bonferroni correction (alpha = 0.0125) in (D). * *p* < 0.05, ** *p* < 0.01, *** *p* < 0.001, **** *p* < 0.0001. In **B** and **G**, * indicates comparisons to FCM-free counterparts and ^ indicates comparison to cocktail-free counterparts

To search for fibroblast-derived niches supporting CD163 expression, we simulated and compared ligand expression in CD163-inductive and non-inductive cocultures with our sc dataset (see “Materials and methods” section; Fig. S5A–B). From the shortlisted ligands, we observed CD163 induction by α2 chain containing laminins, LN211 and LN221 (Fig. 3A, B). Nevertheless, the induction was limited compared to fibroblast-conditioned medium (FCM). The difference was not due to laminin-α2 contents since FCM contained a comparable amount (15.1 ± 5.2 μg/ml) to that in laminin-treated cultures (10 μg/ml). Rather, laminins may act additively or synergistically with other secreted components, such as type-V collagen, to induce CD163 (Fig. 3B–D). FCM immunodepleted for laminin-α2 and any of the associated proteins induced 72.2 ± 4.0% less CD163. Consistently, when native laminin-α2 and type-V collagen, together with any associated proteins, were isolated from FCM, these purified proteins could induce CD163 expression (Fig. 3E). Integrins are well-known receptors for laminins. In the presence of integrin activating Mn^2+^ ions, direct binding of LN211 to macrophages was evident (Fig. S5C). With an ELISA-based interaction assay, we detected both LN211 and LN221 bound integrin β1 (Fig. S5D). Accordingly, blocking integrin β1 by antibody suppressed macrophage adhesion on LN211 (Fig. S5E). Nevertheless, the same blocking antibody failed to interfere with CD163 induction by laminin-α2, suggesting the process may involve a non-integrin β1 receptor (Fig. S5F).

Since S1 macrophage reduced with progressive AD, an inflammatory condition, we tested if inhibiting individual inflammatory pathways induces CD163 expression. We found that pharmacological activity suppression of Syk, Lck/Fyn (Src-family kinases), IKK, STAT5 or BMP signalling stimulated CD163 expression. Amongst these signals, only STAT5 inhibition induced CD163 expression non-additively to FCM, suggesting STAT5 inactivation acts downstream of FCM (Fig. 3F). CD163 induction by FCM required SHP1, which activity suppression reduced the induction by 80.5 (±9.0)%. Inhibition of SHP2 instead induced CD163 expression independently of FCM (Fig. 3G). With SHP1 activity inhibited, CD163 remained inducible by STAT5 inhibition despite a lower magnitude, suggesting a downstream role of STAT5 to SHP1 (Fig. 3H). These results proposed involvement of SHP1 activation and STAT5 inhibition during CD163 induction by FCM. The intrinsic FRβ expression involves down-tuning of STAT6 and type-I TGF-β receptor ALK5 as suppressing these signals strengthened the expression (Fig. 3F). In addition, pathway inference of our sc dataset suggests strong activities of steroid, VEGF, MAPK, p53 and JAK-STAT in S1. However, inhibiting p53 and JNK (a MAPK) instead upregulated FRβ and CD163 expression respectively on FCM-treated macrophages, suggesting these activities are not required to maintain S1 identity (Fig. S6). Hence, intrinsic controls of STAT6 and ALK5 activities during macrophage differentiation coordinate with the fibroblast-triggered SHP1/STAT5 inhibition axis to form FRβ^+^CD163^+^ S1.

### Interplay between AD-upregulated cytokines and laminin availability drives S1 identity loss in AD

Numerous cytokines are upregulated in AD, we explored how these cytokines impact S1 identity by examining their effects on FRβ/CD163 expression. Exposing monocytes to a cocktail of most upregulated cytokines resulted in reduced FRβ expression by 53% (±4%). Intriguingly, when CD163 was induced by secreted factors in FCM, this effect could be prevented (Fig. 3I; Fig. S5G). Assessment of individual cytokines found IL4 and TGFβ3, respectively produced by leukocytes and mesenchymal cells in skin, causing the reduction (Fig. 3J; Fig. S5H). Thus, when fibroblast-derived niches are scarce with reduced CD163 expression on macrophages, AD cytokines act to accelerate S1 identity loss.

**Fig. 4:**
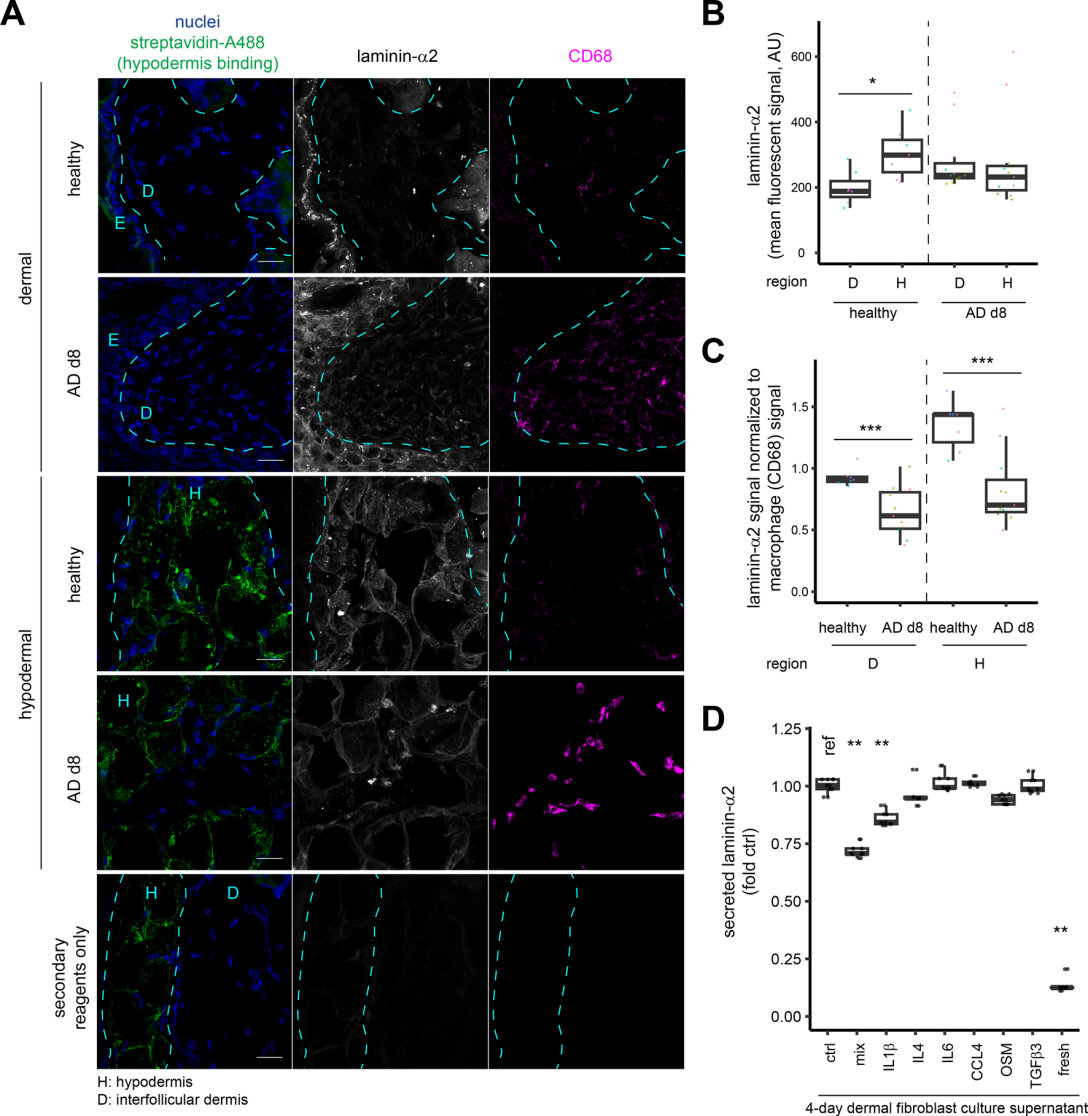
Localization, availability, and regulation of laminin-α2 in AD skin. **A**,**B** Thirty-micron cryosections of healthy or day 8 AD skin were immunostained and quantified for laminin-α2 and CD68 signals in dermis and hypodermis. *n* = 7 or 11 inter-follicular dermal and 7 or 11 hypodermal regions from 3 mice (healthy) or 4 mice (AD). Scales, 20 μm. **C** FCM were prepared in the presence of indicated cytokines. Amounts of secreted laminin-α2 were measured by ELISA and compared to reference. *n* = 10 cultures each from 2 experiments. Groups were compared by *t* test in **B** and **C** or Kruskal–Wallis test with Bonferroni correction in **D**. * *p* < 0.05, ** *p* < 0.01, *** *p* < 0.001

The prerequisite of scarcity of fibroblastic niches for CD163 reduction prompted us to compare the availability of laminin-α2 to macrophages in healthy and AD skin compartments. When immunostained, besides the well-documented localization at muscular basement membrane, we observed laminin-α2 deposition in hypodermis, to an extent stronger than in dermis (Fig. 4A, B). Interestingly, hypodermal laminin-α2 mostly colocalized to type-V collagen, which per se distributed ubiquitously, suggesting macrophage CD163 induction at these laminin-α2^+^ hypodermal sites might be supported by type-V collagen (Fig. 3D; S5H). In AD skin, with immense monocyte extravasation, amounts of laminin-α2 available to CD68^+^ MOMF reduced, (Figs. 1D, 4C) suggesting the CD163-driving capacity required to safeguard S1 identity became limited. Production of de novo laminin-α2 in AD shall also reduce as we found IL1β, an AD-upregulated cytokine, reduced fibroblastic secretion of laminin-α2 in vitro (Fig. 4D; Fig. S5G). This may not be immediately reflected in immunostains due to the relative stability of laminins in tissues. Consequently, reduced CD163 expression on macrophage licensed inflammatory cytokines in AD to further strip S1 macrophage identity.

### S1 macrophages are efferocytic and chemoattract eosinophil and monocyte to skin

**Fig. 5:**
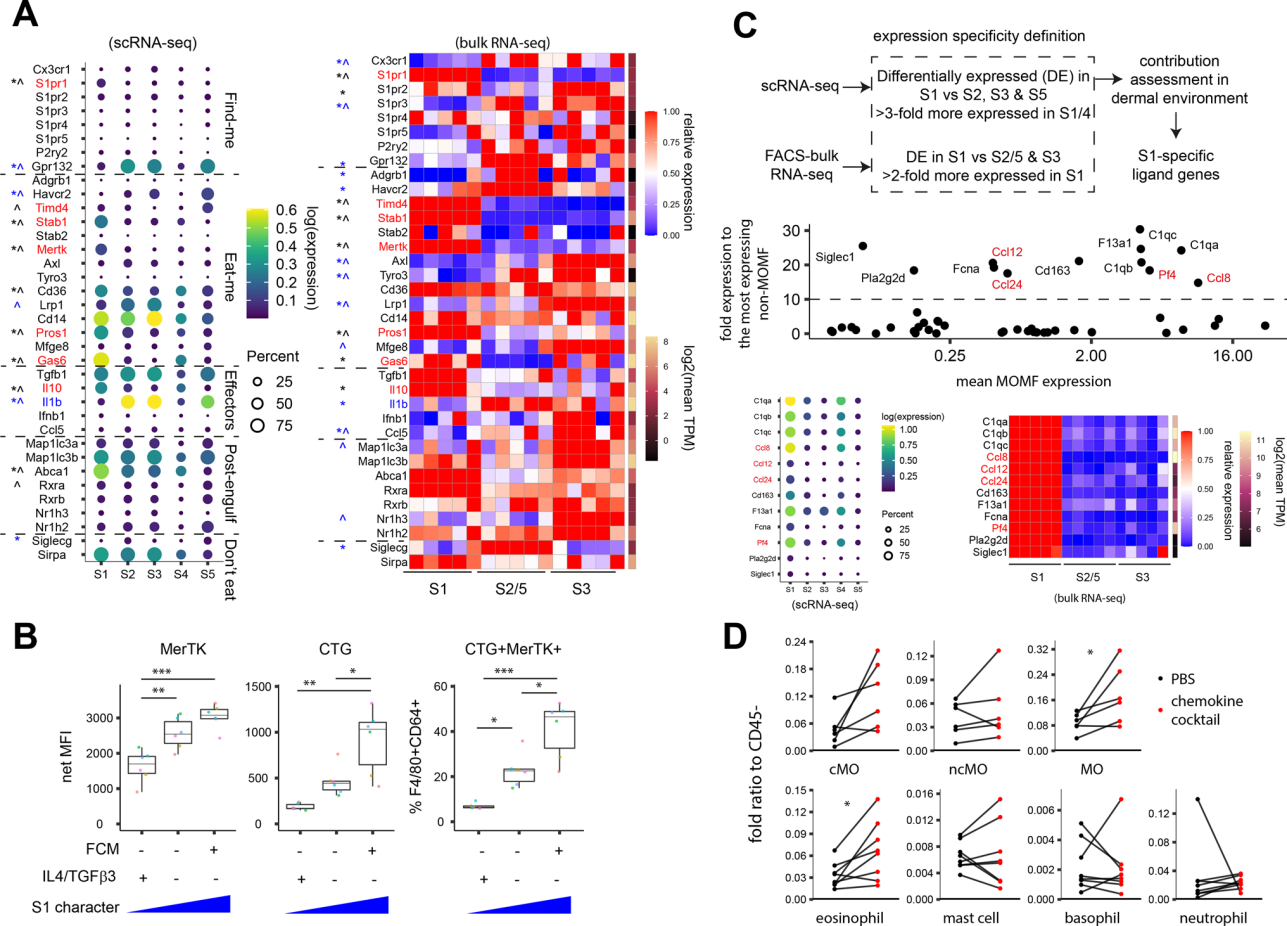
S1 macrophages specialise in efferocytosis and humoral functions. **A** Expression of genes mediating indicated efferocytosis steps were examined in sc and bulk datasets. DEG (*p* < 0.05) of fold-change > 2 versus S2/5 (*) or S3 (^) were indicated. Black, upregulated in S1; blue, downregulated in S1. **B** PB-derived macrophages treated with indicated stimuli were allowed to efferocytose CTG-labelled apoptotic PMN for 2 h. Expression of MerTK and efferocytosis efficiency were measured. *n* = 6 batches (colour-coded) each with cells derived from an individual mouse (biological replicate). **C** Expression of S1(/4)-specific ligand with major contribution in skin were shown. **D** Mice received an intradermal dose of either chemokine cocktail containing 1 μg each of CXCL4, CCL8, CCL12 and CCL24 or PBS on paired lateral regions on back skin. Dermal myeloid leukocytes were analysed after overnight. *n* = 6 mice (monocytes) or 8 mice (others). Groups were analysed by 2-way ANOVA followed by Tukey’s test in **B** or paired *t* test in **D**. * *p* < 0.05, ** *p* < 0.01, *** *p* < 0.001

Inferring functional specialisation of S1 by GSEA, we found S1 being associated with endocytosis and humoral immune functions, and S2/5 relating to leukocyte interaction/activation (Fig. S7A). Molecular profile of S1 indeed suggested a stronger efferocytosis response (higher *Il10*/*Il1b* ratio). Examining the molecule utilised in efferocytosis, S1 showed upregulated *S1pr1* for sensing “find-me” signals; higher expression of phosphatidylserine receptors *Mertk*, *Stab1*, *Timd4* and bridging molecules *Pros1* and *Gas6* to mediate “eat-me” signals (Fig. 5A). Consistently, boosting S1 characters with FCM increased, while dampening S1 characters with IL4/TGFβ3 reduced, MerTK expression on PB-derived macrophages. When the growth surfaces of these macrophages were saturated with apoptotic PMN, more efferocytotic cells were observed in FCM-treatment or ctrl than in IL4/TGFβ3-treatment (Fig. 5B; Fig. S7B). In contrast, while macrophage coculture dampened Th2 skewing, an important aspect of AD, as indicated by reduced GATA3 expression, varying S1 characters of macrophage has no impacts on the phenotype (Fig. S7C). In skin, S1 is the major humoral source of complement system components (*C1qa*, *C1qb*, *C1qc*, *Fcna*), coagulation factor *F13a1*, anti-inflammatory phospholipase A2 *Pla2g2d* [29] and several chemokines (*Pf4*, *Ccl8*, *Ccl12*, *Ccl24*) (Fig. 5C). When this S1 chemokine cocktail was intradermally injected to dorsal skin, amongst myeloid leukocytes examined, we observed eosinophil and monocyte numbers increased by +85% and +73% respectively after overnight (Fig. 5D; Fig. S8). These features suggest potential involvement of S1 in regulating homeostatic monocyte/eosinophil turnover in skin.

### Laminin-α2 recruits S1/4-committed monocyte and homeostatic eosinophil to AD skin

**Fig. 6:**
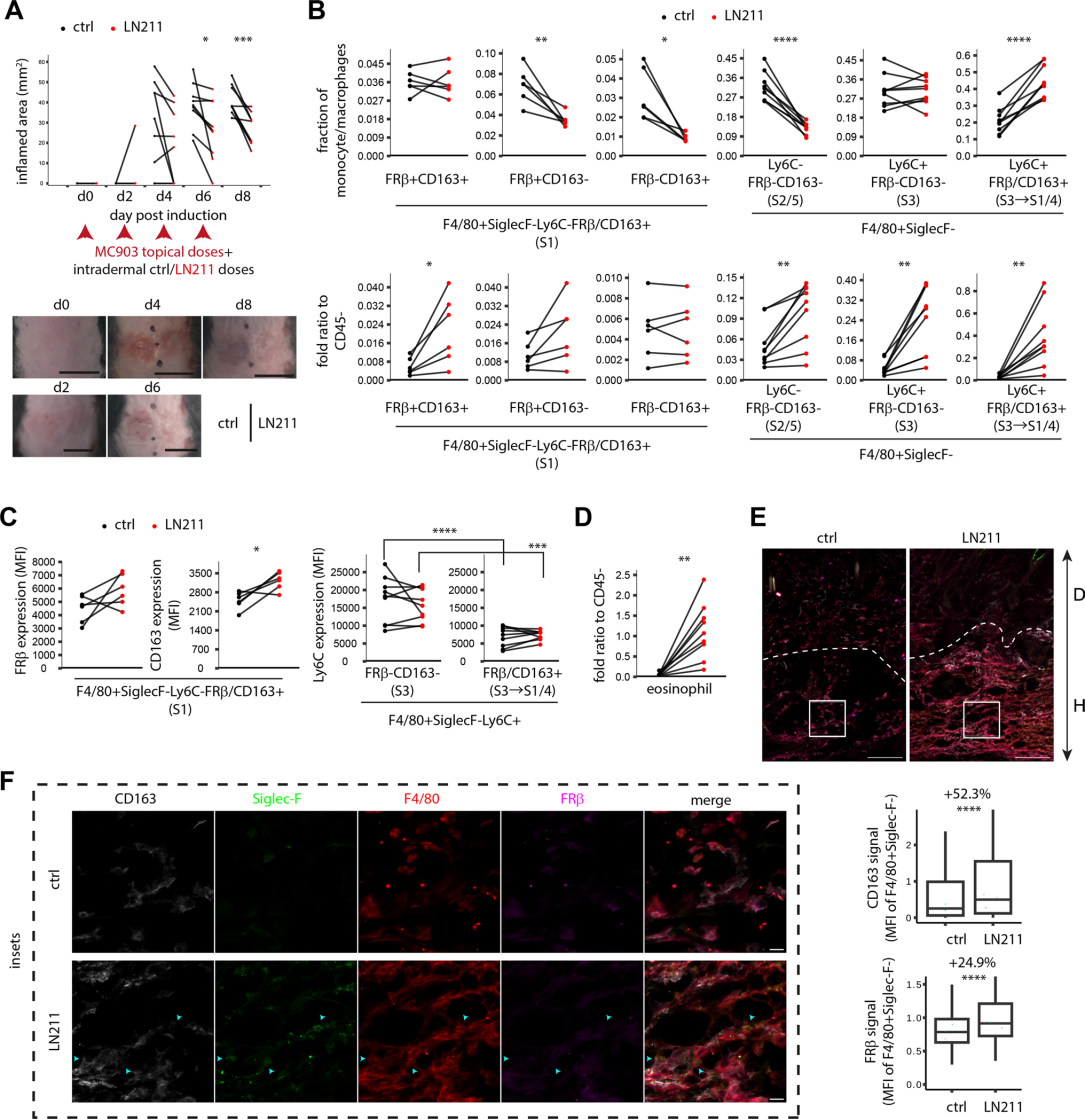
Laminin-211 supports monocyte commitment to S1 and homeostatic eosinophil recruitment in atopic dermatitis. Mice received intradermal doses of vehicle (ctrl) or LN211 after topical doses of MC903 on paired back skin regions. **A** Dermatitis areas were monitored and affected regions were analysed on day 8. Representative paired areas from the same mouse during the course were shown. Scales, 1 cm. MOMF subset proportion (**B**), expression of FRβ, CD163 and Ly6C (**C**) and eosinophils recruitment (**D**) were examined by flow cytometry. *n* = 6 (left panels in **B**, left panels in **C**) or 9 (the rest in **B**–**D**) mice. **E** Expression of FRβ/CD163 on F4/80^+^Siglec-F^−^ in paired dermis (D)/hypodermis (H) regions were examined by volumetric confocal microscopy. Stack projections (23.4-μm) were shown. *n* = 3370 (ctrl) and 5676 (LN211) F4/80^+^Siglec-F^−^ from 6 fields and 3 mice (colour-coded). Colour-coded dots indicated median of each mouse. **F** Localisation of recruited eosinophils in hypodermis were shown (insets of **E**). Cyan arrows indicate S1-bound eosinophils. Images were representative of 3 mice. Scales, 100 μm or 10 μm (inset). White dashed line, D/H interface. Groups were compared by paired *t* test or Wilcoxon signed-sum test followed by Bonferroni correction in **A**–**D** or Mann–Whitney *U* test in **E**. * *p* < 0.05, ** *p* < 0.01, *** *p* < 0.001, **** *p* < 0.0001

We next attempted to restore S1 macrophage niche in AD with CD163-driving LN211 and analysed its consequence. On paired dorsal skin regions, mice received intradermal doses of LN211 or vehicle after every topical MC903 stimulation. LN211 treatment reduced dermatitis inflamed area by 34% (±8%) (Fig. 6A). Proportion of Ly6C^−^ S1 subsets were not directly affected by LN211 despite a slight increase in CD163 expression. However, the S1-committed Ly6C^+^ monocyte fraction increased by +126 (±6)%. Strikingly, intradermal eosinophil increased by +32 (±7)-fold (Fig. 6B–D; Fig. S9). Eosinophil accumulation, considered by some studies as an AD hallmark [30], was unexpected as it did not correlate to a more severe dermatitis. In situ examination confirmed FRβ/CD163 induction on F4/80^+^Siglec-F^−^ MOMF, expansion of MOMF and Siglec-F^+^ eosinophil populations by LN211 (Fig. 6E, F). Both eosinophils and S1-committed MOMF localised to hypodermis, suggesting these processes occurred there (Fig. 6F). Because LN211 presented similar MOMF/eosinophil kinetics against collagen-1, another matrix protein, these leukocyte accumulations were unlikely due to cell adhesion support of matrix proteins (Fig. S10A–D). In addition, when AD skin was treated with type-V collagen, another niche that drives CD163 expression, we observed a similar trend of reduced inflammation. The effect was however weaker than that of LN211 (Fig. S10E). To find molecular features specifically activated when monocytes transit to S1, but not S2, we aligned transiting MOMF by cell state (pseudotime) and isolated S1 transition specific dynamic genes. Co-expression analysis of these genes found two potentially pivotal gene modules specifically and temporally upregulated during S3-to-S1 transition (Fig. S11).

### Laminin-α2 modulates eosinophil phenotype via interaction with S1 macrophage

**Fig. 7:**
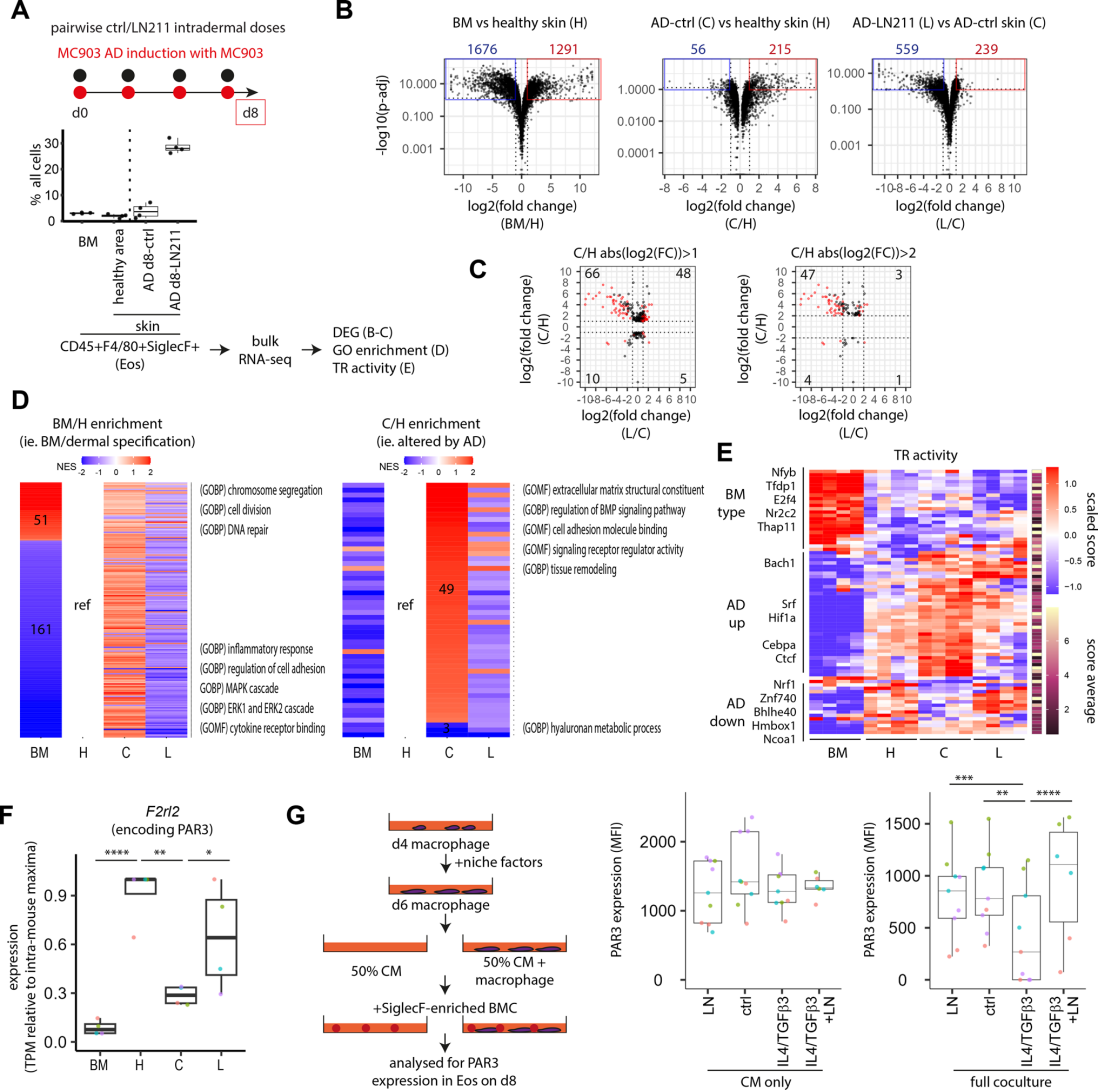
Dermal eosinophil maladaptation during atopic dermatitis was functionally restored by LN211. **A** Schema for bulk RNA-seq analyses of eosinophils FACS-isolated from bone marrow (BM), healthy skin region, vehicle-treated (ctrl) AD skin region and LN211-treated AD skin region of day 8 AD mice as prepared in Fig. 6A. **B** DEG of fold-change > 2 and mean sample TPM > 5 obtained from DESeq2 analysis of indicated sample comparison were shown. **C** Regulation of DEG (left, fold-change > 1, 271 genes; right, fold-change > 4, 118 genes) from C/H comparison by LN211 was shown. Gene numbers in the indicated grid are shown. Red, significant regulation (*p* < 0.05). **D** Enrichment scores of significant (*p* < 0.05) enrichments in reference to healthy skin were shown. **E** Consistently altered (in all four samples) TR activities in BM/H and C/H comparisons were shown with the most active TR labelled. **F** Expression of *F2rl2* in eosinophils of the indicated conditions were shown. *n* = 4 mice for each eosinophil source individually analysed. **G** Schema of eosinophil coculture with PB-derived macrophages treated with indicated niches was shown. PAR3 expression in CD64^−^F4/80^+^Ly6C^+^SSC^hi^ eosinophils was measured by flow cytometry after intracellular staining. *n* = 6 cocultures from 3 mice (IL4/TGFb3/LN211) or 9 cocultures from 4 mice (rest). Each mouse was colour-coded. Groups were compared by DESeq2 in **B** and **F** or 2-way ANOVA followed by Tukey’s test in **G**. * *p* < 0.05, ** *p* < 0.01, *** *p* < 0.001, **** *p* < 0.0001

Accumulation of these apparently non-pathological eosinophils prompted us to scan their bulk transcriptomes for quality shift by LN211 (Fig. 7A; Fig. S12). Analogous to intestinal adaptation of eosinophils recently reported [31], dermal homing of eosinophils regulates a unique subset of genes shifting phenotype from cell cycling to immunomodulation. AD solely upregulated gene expression; only a fraction (43.5%) was reversed by LN211 (Fig. 7B–C). However, at functional level, GSEA analyses indicated many AD-altered gene ontologies, such as ECM reorganization and tissue remodelling, had been restored by LN211 (Fig. 7D). In parallel, bone marrow (BM) eosinophils had more active transcription regulon (TR) [32, 33] involved in biogenesis than dermal counterparts; several TRs playing functional regulatory roles in other leukocytes [34–36] were altered in AD and partially restored by LN211 (Fig. 7E). Hence, instead of direct gene-level reversal, LN211 have reprogrammed eosinophils to achieve a normalized functional phenotype.

Dermal eosinophil PAR3 expression was reduced in AD and restored by LN211 (Fig. 7F). We used PAR3 as an indicator of eosinophil maladaptation to test whether LN211-promoted S1 macrophage is involved in this reprogramming process. Macrophages were treated with IL4/TGFβ3 or IL4/TGFβ3/LN211 to mimic S1 identity loss during AD or the restorative LN211 treatment respectively, and then cocultured with Siglec-F^+^ eosinophil enriched BM cells. Eosinophils cocultured with IL4/TGFβ3-treated macrophages expressed −62 (±12)% less PAR3 which was rescued in cocultures with IL4/TGFβ3/LN211-treated macrophage. In contrast, macrophage secretion did not affect PAR3 expression (Fig. 7G). It thus suggested eosinophil PAR3 loss involved interaction with macrophages of reduced S1 identity which was restored by LN211.

### Gene signature defined identities classify macrophage transcriptomic states in pan-tissue manner

As our macrophage subtype identity definition could detect maladaptation in AD, we further tested whether it applies to detect pathological heterogeneity shifts in other well-documented tissue pathologies. We isolated MOMF of various pathophysiological origins from archived datasets, scored them for subtype characters, and monitored identity projectile following disturbance (Fig. 1C; see “Materials and methods” section). Identity shifts were evident in all conditions examined (Fig. S13; Table S1). For instance, profound S1 identity decline in DSS-induced colitis could be verified by flow cytometry as reduced FRβ+ cells (Fig. S14). *Cd163*/*Folr2* transcripts did not mark S1 in all organs highlighting advantages of using a gene signature for identity definition. To examine how our identity definition correlates to the actual transcriptomic cell state, single-MOMF of various tissue conditions were aggregated to proximity pseudobulks and re-clustered on a common UMAP. Asides from some clusters showing mixed subtype characters, pseudobulks of alike subtype identities shared similar transcriptomic profile and thus tended to converge on UMAP (Fig. S15A; see “Materials and methods” section). This identity definition is hence suitable to describe transcriptomic states of macrophages in a pan-tissue manner.

## Discussion

We here described S1 macrophage as a FRβ/CD163 expression associated subtype supporting steady-state turnovers of apoptotic cells, monocytes, and eosinophils, and documented its depletion as the major maladaptation in progressive AD. This study sought to elucidate mechanisms shaping S1 identity at homeostasis and to determine whether and how restoring this identity impacts AD. We found that intrinsic suppression of STAT6 and ALK5 supported FRβ expression when monocyte differentiated to macrophage. CD163 expression however required signals from fibroblast to suppress STAT5 activity via SHP1. Laminin-α2 and type-V collagen produced by hypodermal fibroblasts was identified to drive macrophage CD163. The driving magnitude was much stronger when both niches were present, and such laminin-α2/type-V collagen dual positive sites were mainly found in hypodermis with laminin being the limiting component. In normal skin where monocyte extravasation is mild [28], these local niches have sufficient CD163-driving capacity to generate S1. Despite exaggerated monocyte extravasation in AD, laminin-α2 was not upregulated and these monocytes received smaller “laminin shares” during differentiation, resulting in lower CD163 expression on macrophages. AD-upregulated cytokines IL4 and TGFβ3 then acted on these CD163^−/lo^ macrophages to diminish FRβ. S1 identity loss in AD is thus a two-step process stemming from the limited S1-driving fibroblastic laminins. This stepwise paradigm is consistent to the observation that FRβ^+^CD163^+^ and keratinocyte-conditioned FRβ^−^CD163^+^, but not the intermediary FRβ^+^CD163^−^, were the major S1 compartments depleted in AD. Accordingly, we demonstrated that modifying the tissue environment in AD skin by increasing laminin-α2 availability exogenously committed monocytes to S1 and reduced inflammation.

Expanded population of eosinophils was additionally observed in laminin-treated skin. Although this fits the chemokine secretion of S1, it is unusual to have eosinophil accumulation without aggravating dermatitis. While this indicated homeostatic nature of these eosinophils, the aberrant recruited number suggested laminin availability is carefully regulated in normal physiology. Although neo-vascularisation in papillary dermis of AD skin has been reported [37], tissue localisation of these eosinophils suggested they have extravasated in hypodermis after laminin treatment. Inferring eosinophil transcriptomes in AD skin suggested functional shifts from the healthy state, marked by PAR3 downregulation, that was partly reverted by laminin treatment. In vitro, eosinophil PAR3 downregulation could be observed after interacting with FRβ/CD163^lo^ non-S1, while cellular interaction with S1, as observed in treated skin in vivo, partially restored PAR3. These findings highlight that, interaction environment and cellular quality of eosinophils determines the functional consequence in tissue and are consistent to their non-allergic presence in various healthy tissues [31, 38].

Alternative to an exogenous supply, the dermal fibroblasts may be targeted to promote their laminin-α2 secretion. To this end, after screening a library of 154 physiological metabolites for compounds supporting laminin-α2 secretion, we identified three natural steroids that boosted basal laminin-α2 secretion and resisted IL1β-induced secretion reduction (Fig. S16). They are intermediates of the cortisol and aldosterone synthesis pathways, and may regulate physiological laminin-α2 secretion from dermal fibroblast. These metabolites nevertheless act on many cell types; fibroblast specificity must be achieved, potentially by chemical modification, before becoming instrumental to promote S1 macrophage.

In the conventional M1/M2 classification system, expression of CD163 on S1 may categorise them as M2-like. However, antagonist roles of S1 in AD contradicted supportive roles of M2 macrophage in AD aggravating Th2/Th22 responses in skin [16]. Analysis of MOMF transcriptomes at single-cell resolution had not detected any subset consistently and specifically expressing M2-marking genes in our dermatitis model. Instead, all MOMF subtypes exhibit mixed M1/M2 profile. The M1/M2 dichotomy has been known to be limited in reflecting in vivo macrophage phenotypes [39]. While both IL4 and TGFβ3 associate with M2 responses [40], we showed that these cytokines and their downstream signals, STAT6 [41] and ALK5 [42], rather suppressed S1-associated FRβ. S1 shall thus be distinguished from M2. Notably, most of the signalling identified to restrain S1-associated FRβ or CD163 are activated in inflammation [43, 44]. Since CD163 expression was critical to guard S1 identity, we have searched and identified relevant signalling pathways. While CD163 induction on macrophages by fibroblastic niches involves STAT5 deactivation, induction could also be achieved by down-tuning Syk, SFK, IKK and BMP signalling. Modulating these other signalling pathways represent potential alternatives to induce CD163-associated S1 identity, yet to be verified in vivo. As we identified α2-laminins and type-V collagens amongst the CD163-inducing fibroblastic niches, and these belong to a bigger family of integrin β1 binding ECM, we have tested whether CD163 induction depends on integrin β1. Nevertheless, the induction process appeared to involve a yet unknown receptor since antibody blockade of integrin β1 binding did not affect the induction by α2-laminins.

Exogenous laminin-α2 converted extravasated monocytes but not local macrophages to S1 in AD. It highlights that the laminin acted at the stage of extravasated monocytes and identity restoration was achieved by guiding their subtype decision. This mode of action implies general applicability as monocyte extravasation is a typical hallmark in many pathologies (enhanced S3 identity). Because the actual identity trajectory depends on the disturbance, tracking the macrophage identity kinetics in pathology-specific manner is essential to direct monocytes to the correct subtype. Unlike AD, S1 identity was instead strengthened in some conditions such as severe and fibrotic forms of kidney bilateral ischemic reperfusion injury and bleomycin-induced pulmonary fibrosis. It has been reported that CD206^+^ macrophages with a transcript expression profile fitting to S1 facilitate fibrosis following renal injuries [45, 46]. Although non-selective suppression of macrophage retarded fibrosis, other subsets with physiological roles were also eliminated [45]. Specific S1 identity reduction could be challenging for the likely presence of multiple maladaptive niches in pathological tissues. Modifying the maladaptive tissue environment by replenishing exhausted niches is thus more leverageable to normalise macrophage identity in general.

Here identity of a macrophage (subtype) is defined by relative expression of signature genes against the population neglecting transcription magnitudes. It requires and presumes a heterogenous macrophage population which is typical in vivo [1, 2, 5]. To our surprise, though the gene signature was constructed with MOMF expression in AD skin, this identity definition was capable to capture cell state changes of MOMF in multiple tissue-conditions, suggesting its general applicability to classify MOMF by transcriptomic cell states. In this identity definition, the well-known inter-tissue variance of macrophage characteristics manifested as varying identity compositions between tissues. It emphasized the presence of macrophages attaining similar cell states despite their distinct tissue origins and residence environment, which had been obscure when MOMF were analysed as averaged tissue-bulks [1, 2]. Compared to other subtypes, MOMF within the S1 category presented the highest inter-tissue variability. In addition to suppressive roles of S1 in AD described in the current study and potential pro-fibrotic roles following pulmonary and renal injuries mentioned above, transcription regulons indicative of S1 (Supplementary Data S1), concordantly present diverse functional roles in tissues. While Ets2 supported general inflammatory responses in tissue macrophages [47], Ets2 in mammary TAM did not elicit antitumoral responses but promoted tumour angiogenesis [48]. Xbp1 in lung promoted but Esr2 in skin suppressed inflammation [49, 50]. Nfe2 activity in macrophages appears crucial to matrix remodelling in brain [51]. Hence, functional variance of macrophage in different tissues may be attributed to distinct identity compositions, qualities of S1 as well as different crosstalk to local tissue cells.

Collecting public single-cell transcriptomes of macrophages from 10 organs and 12 perturbations, Macrophage Identity Kinetics Archive (MIKA) is a pseudobulk-based pan-tissue universe aiming to record all possible in vivo macrophage states with retrievable information, such as tissue origin, gene expression and guiding niches, registered to each cell state (Fig. S15A). Of note, expression of *Folr2* and *Cd163*, markers of the described dermal S1 macrophages, are restricted to a focal region in this pan-tissue space, proposing that FRβb/CD163^+^ macrophages as a transcriptionally homogenous population even across different tissues (Figure S15B). This however does not necessarily equate functional homogeneity, which depends on the interaction context in the tissue environment. Adopting the S1–S5 subtype identity scheme, MIKA could detect identity changes in pathologies (Figure S13). This pan-tissue universe could further expand by incorporating emerging datasets. Mapping macrophages of interest to MIKA could inform whether the macrophage is similar to any archived cell state, whether a specific surface marker gene presents, and whether there is any previously identified guiding niche. MIKA is thus expected to enable sophisticated control of macrophage identity for pathology restoration in the future.

## Materials and methods

### Animal procedures

Female C57BL/6 mice aged between 7- and 14-weeks old were used for experiments. Atopic dermatitis induction by topical MC903 treatment (MCE, HY-10001, dissolved in ethanol) was previously described [4]. In some experiments, PDGFRα-H2BGFP knock-in reporter mice [52] were used. In some experiments, mice received intradermal doses of vehicle (containing 10% glycerol and 0.02% NaN3 in PBS), 5 μg of either collagen-1 (Corning, 354236) or LN211 (Biolamina, LN211-0501) or type-V collagen (Sigma, CC077) 10-min after each topical MC903 doses. Weekly schedule of 10–5-5-nmol MC903 was adopted if AD was induced on only one region; 5–5-5-nmol weekly schedule was adopted if paired regions were induced. Dermatitis was monitored by measuring areas of inflamed eclipse by 0.25π (width) x (length), as shown in Figs. 1A, 6A, and S1B. To examine leukocyte recruitment by S1 chemokines, a cocktail containing 1 μg each of CXCL4, CCL8, CCL12 and CCL24 (250-39, 250-14, 250-04, 250-22, all from PeproTech) or PBS was intradermally delivered to dorsal skin. Treated areas were harvested for analysis after overnight. For preparing homeostatic cryo-samples, both genders were used. Animals were maintained in a barrier facility under special pathogen-free conditions. All animal experiments were conducted under the guidelines of and approved by the Institutional Animal Care and Use Committee of Osaka University Graduate School of Medicine with reference numbers 02-075-003 and 02-075-005.

### Cell culture

To differentiate PB monocytes to macrophages, heparinised blood was obtained from inferior vena cava (IVC) with erythrocytes lysed at room temperature for 10 min. Cells from 70 to 100 μl blood volume equivalent were cultured in a collagen-I-coated well of 12-well plate in RPMI containing 20 ng/ml M-CSF (PeproTech, 315-02), 10% FBS, 1% glutamine and 1% penicillin–streptomycin (PS) (differentiation medium) for 4 days. Medium was either refreshed on day 4 or shifted to coculture medium (SMC medium (Sigma, 311F-500) with 20%FBS, 20 ng/ml M-CSF, 12 ng/ml FGFβ (PeproTech, 450–33), 5 ng/ml IL2 (Biolegend, 575402), 56 μM β-mercaptoethanol (Nacalai Tesque), 1%NEAA (Nacalai Tesque), 1% glutamine and 1% PS) with or without addition of other cells or reagents for another 4 days. To isolate T cells from PB, erythrocyte-lysed heparinised blood was blocked with 10 μg/ml 2.4G2 in PBS with 2% FBS and then incubated with 2 μg biotinylated anti-CD3e (Biolegend, 145-2C11) per mouse on ice for 20 min followed by washing. Cells were incubated with 10 μl per mouse MojoSort-streptavidin beads (Biolegend, 480016) at 4 °C for 20 min under rotation followed by magnetic isolation for immediate uses. Primary mouse endothelial cells (MDMVEC) were isolated from tail dermis as described previously [53]. Dermal fibroblasts were isolated from excised dorsal skin digested in DMEM containing 0.3% collagenase-A (Roche), 0.25 mg/ml dispase-II, 10 U/ml DNase-I and 2% FBS at 37 °C for 1 h with agitation. Cells passed through 70-μm strainer were cultured in DMEM containing 10% FBS, 1% glutamine and 1% PS. Smooth muscle cells were isolated from excised IVC and digested as described above. Cells were expanded in SMC medium supplemented with 5%FBS, 1% glutamine and 1% PS. To isolate keratinocytes from tail epidermis, peeled tail skin was digested in DMEM containing 0.25 mg/ml dispase-II and 2% PS at 37 °C for 1 h with agitation. Epidermis sheet was separated from dermis and further digested in accutase at room temperature with agitation for 20 min. Released cells passing through 70 μm-strainer were expanded in Keratinocyte Growth Medium 2 with complete supplements and 0.06 mM CaCl_2_ (PromoCell, C-20011). Leukocytes were never passaged. MDMVEC was used at <10 passages. Other cells were used at <5 passages.

### Antibodies and reagents

The following primary antibodies were used for flow cytometry and confocal microscopy (all from Biolegend): anti-CD163-BV421 or -APC (S15049I) and anti-FRβ-APC (10/FR2). The following primary antibodies were used for flow cytometry (from Biolegend if unspecified): anti-F4/80-FITC or -BV421 (BM8), anti-CD163-PECy7 (S15049I), anti-Ly6C-PE (HK1.4), anti-SiglecF-PECy7 or -PE (S17007L), anti-CD45-FITC or -PECy7 or -PerCP-Cy5.5 (30F11), anti-MHCII-Alexa-Fluor(AF)-488 (M5/114.15.2), anti-CD49b-APC (HMa2), anti-FceR1a-PECy7 (MAR1), anti-CD117-PE (2B8), anti-VE-Cad-BV421 (BV13), anti-PECAM-1-APC (390), anti-MerTK-APC (2B10C42), anti-PAR3-AF647 (G4; Santa Cruz, sc393127), anti-CD4-APC (GK1.5), anti-GATA3-BV421 (16E10A23), Annexin-V-PerCP-Cy5.5, 7AAD and Sytox-blue. The following antibodies were used for confocal microscopy (from Biolegend if unspecified): anti-PDGFRα (abcam, ab203491, EPR22059-270), anti-PDGFRα-biotin (APA5), anti-E-Cad-AF-594 (DECMA1), anti-F4/80-AF-594 (BM8), anti-Siglec-F-AF-488 (S17007L), streptavidin-AF-488, anti-laminin-α2-AF-546 (abcam, ab11576, 4H8-2; AF fluorochrome inhouse-coupled), anti-CD68-AF-647 (FA11) and mouse-anti-APC-Alexa-fluor-647 (R&D Systems, FAB8927R). Antibody specificity to cellular entities was verified in Fig. S17 with nuclear counterstain or co-staining of the intracellular MOMF marker CD68. The following antibodies were used in ELISA at indicated concentrations: 10 μg/ml mouse-anti-rat-IgG (Fc specific, Jackson Laboratories, 212–005-104) (coat), 1 μg/ml anti-laminin-α2 (abcam, ab11576, 4H8-2) (capture) and 1 μg/ml anti-pan-laminin (abcam, ab11575) (detect). The following reagents (aqueous if unspecified) were used in cell culture at indicated concentrations: 400 ng/ml Sema3c (1728-S3), 400 ng/ml chemerin (2325-CM-025/CF), 5 μg/ml rh-fibrillin (10224-FI-050), 40 ng/ml rm-OSM (495-MO/CF) (all from R&D Systems), 10 μg/ml human type-V collagen (Sigma, CC077), 100 μg/ml sodium hyaluronate (Nacalai Tesque, 18237–41), 10 μg/ml human LN211 (Biolamina, LN211-02), 10 μg/ml human LN221 (Biolamina, LN221-02), 30 μg/ml anti-integrin β1 (HMβ1-1), 10 μM pifithrin-α HBr (p53 inh., S2929), 3 μM ruxolitinib phosphate (JAK1/2 inh., S5243), 5 μM BMS-582949 (p38 inh., S8124), 10 μM FR180204 (ERK inh., S7524), 10 μM SP600125 (JNK inh., S1460), 2 μM IKK16 (IKK inh., S2882), 1 μM RepSox (ALK5 inh., E616452), 10 μM PP2 (Lck/Fyn inh., S7008), 1 μM PRT060318-HCl (Syk inh., S7738), 5 μM BD750 (STAT5/JAK3 inh., S0981), 1 μM AS151499 (STAT6 inh., S8685), 0.5 μM RMC4550, 1 μM SHP099HCl (SHP2 inh., S8718 and S8278), 1 μM NSC87877 (SHP1/2 inh., S8182) (all in DMSO and from Selleck), 2 μM LDN193189-HCl (ALK1/2/3/6 inh., Sigma, SML0559, in DMSO), 40 ng/ml rm-CCL4 (250–32), 40 ng/ml rm-IL1β (211-11B), 40 ng/ml rm-IL4 (214–14), 40 ng/ml rm-IL6 (216–16) (all from Peprotech), 40 ng/ml rh-TGFβ3 (Biolegend, 585802).

### Depletion or isolation of components from FCM

FCM were prepared by culturing ten thousand dermal fibroblasts in collagen-1 coated 12-well plate in 1 ml coculture medium for 4 days followed by supernatant harvest. To deplete FCM for LNa2, a microcentrifuge column was packed with 250 μg anti-LNa2 covalently immobilized on agarose resins (Santa Cruz, sc59854AC) and equilibrate in PBS before use. One millilitre of FCM was allowed to pass through the column followed by 3-times PBS wash and elution with 100 mM Glycine–HCl pH2.2. After elution, the column was re-equilibrated in PBS. Depletion was repeated with the flow-through for five more times to achieve >90% reduction of LNa2 amount when measured by ELISA. To isolate LNa2 and associated components from FCM, eluent was neutralized with one-tenth volume of 1 M Tris–HCl pH8.0 and dialysed against PBS (>one-million-fold dilution). Protein concentrations in dialysed eluents were measured by a NanoDrop spectrophotometer. To isolate Col5 and associated components from FCM, anti-Col5a1 covalently immobilized on agarose resins (Santa Cruz, sc166155AC) were used.

### Volumetric confocal microscopy and analyses

Excised dorsal skin samples were fixed in 4%PFA under gentle pressure at room temperature for 2 h and embedded in OCT compound (Sakura Finetek Japan). One-hundred-micron cryosections were prepared for immunostaining as described previously [4]. Volumetric image stacks were acquired with LSM880 or LSM980 (Zeiss) under 40× objective lens. Spectral unmixing was performed on Online Fingerprinting mode. To quantify FRβ expression on CD163+ macrophages, isosurface objects were built from CD163 signals by IMARIS (Oxford Instruments, v9.9.1) and classified by anatomical positions. FRβ expression was measured as mean fluorescent intensity (MFI) normalized to population maxima. To quantify laminin deposition in skin (30-micron cryosections), surfaces were manually built from nuclei and streptavidin-AF488 signals and used as masks to quantify CD68 and laminin-α2 signals in inter-follicular dermal or hypodermal regions. To quantify FRβ/CD163 on F4/80^+^Siglec-F^−^ cells in ctrl/LN211-treated-in-pair samples, a volume of interest centring at the dermis/hypodermis interface and spanning across the dermis and hypodermis for ~ 600 μm was used since the frequency and distribution of macrophages differ by the dermal depth. F4/80^+^ isosurface were constructed with watershedding within the ROI and Siglec-F^−^ volumes between 50 and 1000 μm^3^ were quantified.

### Cellular binding assay

Macrophages (BMMF) were obtained by culturing one femur/tibia equivalent of bone marrow cells (BMC) in differentiation medium for 4 days. Cells were dissociated by accutase and resuspended at 3 × 10^6^/ml in resuspension buffer (RB, 10 mM HEPES buffered at pH7.4 in DMEM) for assessment of laminin binding with a modified method as previously described [53]. Laminin-antibody complex was prepared by incubating 0.5 μg LN211, 0.25 μg anti-laminin-α2 (abcam, ab11576, 4H8-2), 0.125 μg donkey-anti-rat-IgG(H + L)-AF647 (Invitrogen, A48272) in 25 μl RB containing 0 mM or 1 mM MnCl_2_ at room temperature in the dark for 15 min. In control samples, laminin was omitted. Twenty-five microlitre cells were incubated with 25 μl laminin-antibody complex at room temperature in the dark for 30 min. Cell mixture was then fixed in 1% PFA for 8 min, washed and analysed for bound complex by flow cytometry.

### Interaction ELISA

Ninety-six-well ELISA plate was coated with 10 μg/ml LN211 or LN221 at 4 °C for overnight. Plate was blocked in 1% casein at room temperature for 1 h, washed trice and incubated with 50 μl per well BMMF lysates for overnight. BMMF cultured on a 90 mm-plate was scraped and lysed in 1 ml lysis buffer (Thermo Fisher Scientific, 87787) containing 2 mM each of MgCl_2_, CaCl_2_ and MnCl_2_, and protease/phosphatase inhibitors at 2× concentration (Thermo Fisher Scientific, 78443) followed by incubation at 4 °C under constant rotation for 1 h. Lysates were centrifuged to remove the insoluble fraction. ELISA plate was washed trice with PBS containing 0.05% Tween-20 and 1 mM MnCl_2_ and incubated with 50 μl per well primary antibodies (1 μg/ml anti-integrin β1 (R&D Systems, AF2405), 100× diluted anti-integrin β2 (Cell Signaling Technology, E9O7W, 72607), 0.66 μg/ml anti-integrin β3 (Cell Signaling Technology, D7X3P, 13166) or 3.38 μg/ml anti-integrin β5 (Cell Signaling Technology, D24A5, 3629)) at 4 °C for overnight. Plate was washed trice and incubated with secondary antibodies at room temperature for 1 h, washed quadruple and developed for colorimetric signals (Abs (450 nm)–Abs (570 nm)). Control wells without primary antibody incubation were used to estimate background levels.

### Efferocytosis assay

PMN were prepared from BMC in femur/tibia and labelled with Cell Tracker Green (CTG, Thermo Fisher, C7025) as previously described [53]. Apoptosis was induced by culturing PMN in growth factor deficient medium (RPMI with 10%FBS, 10 mM HEPES at pH7.4, 1% glutamine and 1% PS) for overnight. Apoptotic PMN (aPMN) were calculated from viable counts (VC) and percentages of CTG^+^Sytox^−^ (pV) and CTG^+^annexin-V^+^ (pAV) particles within CTG^+^ by (VC/pV x pAV). To bypass chemotaxis and specifically measure efferocytosis, growth surfaces of blood monocyte derived macrophages were saturated with 50,000/cm^2^ aPMN and incubated at 37 °C for 2 h. Non-interacting aPMN were washed twice and cells were dissociated by accutase for flow cytometric measurement of CTG^+^ macrophages and MerTK expression.

### Eosinophil coculture

Macrophages were prepared from PB monocytes by culturing 20–30 μl blood volume equivalent of erythrocyte-lysed suspension on a collagen-I-coated well of 96-well plate in differentiation medium as described. On day 4, cells were treated with soluble niches (indicated in figures) in coculture medium. On day 6, BMC from femur/tibia were enriched for Siglec-F^+^ eosinophils by staining with anti-Siglec-F-biotin (S17007L; Biolegend, 155512) and positive selection with streptavidin nanobeads (Biolegend, 480016). Half of the macrophage conditioned medium (CM) was transferred to a new well. Twenty thousand eosinophils were then cocultured in 50% CM with or without macrophages. On day 8, cocultures were dissociated by accutase and analysed for PAR3 expression in CD64^−^F4/80^+^Ly6C^+^SSC^hi^ eosinophils by flow cytometry following fixation and intracellular staining (Biolegend, 421002).

### T cell coculture

Coculture experiment was performed as described in eosinophil coculture with the following modification. Anti-CD3e isolation bead enriched naïve T cells from peripheral blood were used. On day 6, Forty thousand T cells were cocultured in 100% CM in the presence or absence of macrophages. Cells were activated by adding T cell activation beads (Thermo Fisher, 11452D) to 1:1 ratio to the number of T cells. On day 8, intracellular flow cytometry was performed to measure GATA3 expression in F4/80^−^CD4^+^ Th cells.

### Transcriptomics

For single-cell RNA-sequencing, skin digests were blocked for Fc receptor by 2.4G2 and enriched for CD45^+^ leukocytes with anti-CD45 magnetic nanobeads (Biolegend, 480028) followed by library preparation with Chromium Single Cell 3′ Reagent Kits (v3.1 Chemistry; 10× Genomics). The 3′ gene expression libraries with paired-end Read 1 (containing 16-bp barcode and 12-bp UMI), Read 2 (91-bp insert) and i7 index (8-bp) were analysed by Illumina HiSeq X (CoMIT Omics Center, Graduate School of Medicine, Osaka University). Expression matrices were constructed from Fastq files after aligning to *Mus musculus* mm10 reference and aggregating samples by CellRanger (v4.0.0). All samples passed the default quality standards of CellRanger. Cells with mapped genes ranging between 200 and 5000 and less than 10% mitochondrial genes were analysed with Monocle3 [54]. Batch effect was removed by batchelor [55]. A MOMF cluster on UMAP was defined by detection of *Csf1r* or *Adgre1* in >50% cells or *Cd68* in >60% cells or *Cd207* in >40% cells. Expressed genes were defined by being detected (UMI ≥ 1) in at least 10% of a cell type or a MOMF subtype.

To measure subtype characters in single-cell macrophage transcriptomes given a list of subtype-specific signature genes (Supplementary Data S2), for every cell, each signature gene expression was first scaled across the whole population to equalise contribution and then averaged to obtain a score for the concerned subtype. Scores for each subtype were scaled by population to fit into the range of 0–1. Since the algorithm relies on relative expression of signature genes across the population, subtype scores are sensitive to input cell population and kinetics shall be monitored with samples isolated in the same manner. Macrophages are preferentially isolated in silico from total cells or inclusive enrichment to preserve heterogeneity for accurate subtype character measurement (Fig. S1A). MOMF were isolated from GEO archived datasets by applying the described criteria without *Cd207* consideration.

To simulate coculture experiment with the sc dataset containing about one-third non-hematopoietic cells, ligands with expressed receptors on macrophages were extracted from annotated database [56] and expression were summarized by cell types. Ligand expression of cell types present in a coculture were summed to estimate the ligand contents. Ligand contents in cocultures which do (positive coculture) or do not (negative coculture) induce CD163 expression on macrophages were compared by *t* test. Ligands of significantly high expression in positive coculture than in negative coculture (fold-change >3 and *p* < 0.05) were shortlisted.

For bulk RNA-sequencing, RNA was extracted from sorted cells and purified with RNeasy Mini Kit (Qiagen) followed by library preparation as previously described [4]. Genes with sample mean TPM > 5 were considered as expressed. Differential analysis was performed with DESeq2 [57]. TR and pathway activities were inferred from log-normalized expression data with decoupleR by DoRothEA and PROGENy respectively [58, 59].

To visualise the transcriptomic landscape and subtype characters of MOMF isolated from GEO archived datasets (described above), pseudobulks from each tissue-condition were first constructed, integrated based on commonly detected genes, reclustered and then projected on UMAP. In each dataset where individual UMAP is constructed, cells in a sample representing a tissue-condition were summarised to 100 pseudobulks by iterating UMAP proximity pseudobulking. A pseudobulk was constructed by aggregating transcripts from its proximal cells with a random seed cell on the UMAP (number of cells being aggregated, or pseudobulking factor, defined as the rounded-up value of total number of cells in a sample divided by 100). Seed cells were randomly selected from unaggregated cells during the iteration to abstract the UMAP. This UMAP proximity pseudobulking process aggregates cells of similar transcriptomes to improve genomic coverage of the resulted pseudobulks, reduces cell number difference between datasets and computational burdens. Subtype characters (scaled scores fit to the range of 0–1 described above) of the resulted pseudobulks are averages of the constituent cells. Sample-wise batch effect was removed by batchelor before reclustering [55].

### Statistics

Data normality was verified by Shapiro–Wilk test. Two-tailed Student’s *t* test (normally distributed, ND) or Mann–Whitney *U* test (non-normally distributed, NND) was used to compare two groups. For paired tests, paired *t* test (ND) or Wilcoxon signed-sum test (NDD) was used. One-way ANOVA (ND) or Kruskal–Wallis test (NDD) followed by a post-hoc test and Bonferroni correction was performed for multiple comparison. In some experiments, two-way ANOVA were used to account for inter-batch variance. Statistical significance was considered at α-level of 0.05.

## Supplementary Information

Below is the link to the electronic supplementary material.

Supplementary file1 (DOCX 17 KB)

Supplementary file2 (XLSX 16 KB)

Supplementary file3 (XLSX 30 KB)

**Supplementary Figure S1: Dynamics of macrophages and other leukocytes during atopic dermatitis.** (A) Leukocyte composition in AD progression was shown. Monocyte/macrophages were isolated from the sc transcriptomes for downstream analyses. (B) Inflamed area were monitored following AD induction with MC903 on back skin of PDGFRα-H2BGFP knock-in mice. n = 14 mice for d0, d2 and d4 or 17 mice for d7. (C) Fold ratio of indicated cells to PDGFRα+ stromal cells in healthy/AD skin regions paired within the same mouse was measured by flow cytometry. n = 6 mice for mast and basophil; n = 7 mice for leukocyte, eosinophil and endothelial cell. Data described in Fig. 1D-F were analysed for (D) expression specificity on MOMF, eosinophil and stromal cells or (E) marking specificity of CD163 and FRβ for F4/80+ macrophages in healthy or AD skin. Groups were compared by paired t-test or Wilcoxon signed-sum test in (C). *p<0.05, **p<0.01, ***p<0.001. **Figure S2: Gating strategy for identification of stromal cells, granulocytes and macrophage subtypes.** Identification of (A) mast cells, basophils and (B) eosinophils for fold ratio quantification to PDGFRα+ stromal cells in Fig. S1C were shown. In (B-C), identification of macrophage subtypes quantified in Fig. 1D-F were shown as (I-VI). **Figure S3: Expression of CD163 and FRβ in time course during PB monocyte differentiation.** (A) Gene expression of CSFs in skin were examined in the indicated cell types with sc transcriptomes. (B) PB monocytes were differentiated with 20ng/ml M-CSF for the indicated period followed by flow cytometry measurement of CD163 and FRβ expression on F4/80+CD64+ macrophages. n = 4, 8 and 6 batches for d4, d6 and d8. (C) Differentiating PB monocytes were cocultured with the indicated cell mixture as described in Fig. 2B for 6 days. n = 6 batches for ctrl and complete coculture (all), and n = 4 for the rest. Each batch is derived from an individual mouse; sample marked with (ref) served as batch-specific internal ctrl. Error bars, SEM. Groups were compared to batch-specific ref by 2-way ANOVA followed by Dunnett’s test in (C). *p<0.05, **p<0.01. **Figure S4: FRβ expression on S1 macrophages differ by dermal depth in vivo.** (A-B) Hypodermis was recognised by endogenous binding of streptavidin (SA) and FRβ expression on CD163+ macrophages of different anatomical locations in healthy skin were quantified with volumetric confocal 3D-stacks and IMARIS. Purple border, E-Cad+ follicular keratinocyte (fKC) interacting; cyan border, dermal; yellow border, hypodermal. n = 172 fKC-interacting, 1113 dermal and 736 hypodermal CD163+ cells from 10 mice. Scales, 50um (wide-fields) or 10um (insets). Colour-coded dots indicated median of each mouse. Groups were compared by Kruskal-Wallis test with Bonferroni correction in (B). *p<0.05, ***p<0.001, ****p<0.0001. **Figure S5: Bioinformatics simulation predicts S1 identity modulating soluble niches.** (A-B) Coculture experiments described in Fig. 2 were simulated with expression data obtained from single-cell transcriptomes. Ligand candidates derived from fibroblast with significantly higher (p<0.05) expression in CD163-inductive cultures were shortlisted for experimental verification (red). (C) Binding of LN211 to bone marrow derived macrophages (BMMF) were measured in the presence or absence of MnCl2. n = 4 batches each. (D) Immobilized LN211 or LN221 were allowed to interact BMMF lysates and detected for indicated integrin binding by ELISA. n = 8 interaction wells with separate lysates from 4 individual mice. Backgrounds (dotted lines) were estimated by primary antibody omission. (E) BMMF were treated by 30ug/ml of the indicated blocking antibodies and allowed to bind on LN211-coated surface (non-tissue culture treated) in the presence of 40ng/ml CCL2. Adherent cells were enumerated. n = 16 wells from 4 mice. (F) CD163 induction by laminins were tested in the presence of the indicated antibodies. n = 4 mice. (G) Single-cell transcriptomes were examined for cell type-wise cytokines expression in indicated conditions. Top 15 significantly upregulated (q<0.05, ranked on expression and fold-change) cytokines on d8 were shown. (H) Thirty-micron cryosections of healthy skin were immunostained and examined for distribution of collagen-V and laminin-α2 in hypodermis. Both stack projection and single-slice images (for colocalization view) were shown. Images were representatives of n = 4 mice. Scales, 50um. Groups were compared by t-test in (B, E), or one-sample t-test in (C-D), or one-way ANOVA followed by Tukey’s test in (F), or Monocle3 gene-fit model in (G). **Figure S6: Core pathway activities in dermal macrophage subtypes.** (a) Dermal MOMF sc transcriptomes described in Fig. 1B were computed for 14 core pathway activities defined by the PROGENy model. (B) Pearson correlation to subtype character score were analysed. (C) FRβ/CD163 modulation capacity of FCM was assessed as described in Fig.3 in the presence of indicated signalling inhibitors. n = 6 batches each. Each batch (colour-coded) is derived from an individual mouse. Groups were analysed by 2-way ANOVA and compared to reference sample by Dunnett’s test. *p<0.05, ***p<0.001. **Figure S7: Functional enrichment analyses on dermal macrophage subtypes.** (A) Gene ontologies (GO) associated to each macrophage subtype were identified by cross-validating single-cell and bulk transcriptomes described in Fig. 1A-C. For each subtype, positively enriched GO biological processes (GOBP) or molecular functions (GOMF) over any other subtype consistently found in bulk and sc datasets are shown. NES, normalized enrichment score. Black, p<0.05; grey, p>0.05. (B) Gating scheme for Fig. 5B to identify efferocytic macrophages under indicated treatment. (C) Peripheral CD3e-enriched naïve T cells were stimulated by anti-CD3/anti-CD28 activation beads in the presence or absence of macrophages of varying S1 character. Expression of GATA3 was measured by intracellular flow cytometry. n = 6 cocultures (3 T cell batches and 2 macrophage batches). Groups were compared by 2-way ANOVA followed by Tukey’s test. ****p<0.0001. **Figure S8: Gating strategy for identification of dermal myeloid leukocytes.** Identification of neutrophil, eosinophil, mast cell, basophil, classical and non-classical monocytes for quantification in Fig. 5D were shown. **Figure S9: Gating strategy for identification of dermal macrophage subtypes after LN211 inoculation.** Identification of MOMF subsets (I-VI) in treated WT mice, corresponding to those marked in Fig. S2B-C, for quantification in Fig. 6B were shown. **Figure S10: Homeostatic leukocyte accumulation induced by LN211 in atopic dermatitis skin was not due to matrix retention.** Experiment described in Fig. 6 was repeated with mice receiving intradermal doses of collagen-1 (Col1) or LN211 after topical doses of MC903 on paired back skin regions. (A) Dermatitis progress was monitored and affected regions were analysed on day 8 for (B) MOMF subset proportion, (C) expression of FRβ, CD163 and Ly6C and (D) eosinophils recruitment. n = 6 mice. (E) Experiment was repeated to compare effects of intradermal doses of vehicle (ctrl) or type V collagen (Col5). n = 8 mice. Scale, 1cm. Groups were compared by paired t-test or Wilcoxon signed-sum test followed by Bonferroni correction. *p<0.05, **p<0.01. **Figure S11: Activation of gene modules specific to S1-commitment of monocytes.** Pseudotime were computed for trajectories to S1 or S2 with S3 root using dermal MOMF sc transcriptomes described in Fig. 1B. Genes fluctuated by pseudotime were identified for each trajectory. S1 transition specific fluctuating genes with temporal expression peaks (within 0.25-0.75 relative pseudotime range) were identified and analysed for inter-gene coexpression. Members of the indicated gene modules were shown. **Figure S12: Effects of LN211 on dermal eosinophil transcriptome.** AD was induced on a paired region of back skin which received either vehicle or LN211 inoculation after each MC903 dose as described in Fig. 6A. On day 8, eosinophils (higher granularity compared to F4/80+SiglecF- MOMF) were FACS-isolated from the indicated tissues for bulk transcriptome analyses described in Fig. 7. **Figure S13: Subtype classification and proportional dynamics upon pathophysiological perturbation of macrophages isolated from publicly archived dataset.** MOMF were isolated from the indicated datasets as Ptprc+Csf1r+Adgre1+Cd68+ clusters (annotated with the corresponding accession). Cells were assessed for macrophage subtype identities with signature genes described in Fig. 1C. Subtype identity dynamics upon indicated perturbation were evaluated. **Figure S14: Subtype dynamics of MOMF during DSS-induced colitis verified S1 decline.** Male mice were given 1.5% DSS-containing drinking water ad libitum for 6 days. On d0 and d6, colons were analysed by flow cytometry for F4/80+MHCII+ MOMF subset proportion. n = 4 (d0) or 3 (d6) mice. Error bars, SEM. Groups were compared with 2-tailed unpaired t-test. *p<0.05, **p<0.01, ***p<0.001. **Figure S15: Pan-tissue atlas of macrophage transcriptomic cell identity states.** (A) MOMF from various tissue-conditions described in Table S1 were summarised as pseudobulks (n = 5300 pseudobulks) and mapped to a single UMAP. Distribution of subtype identities in each organ and between organs were examined. (B) Expression of dermal S1 marking Folr2 and Cd163 across macrophages in pan-tissue context. **Figure S16: Cholesterol derivatives promote fibroblast secretion of laminin-α2.** (A) Dermal fibroblast was cultured with various natural metabolites (tested as 100-fold dilution of the stock) in a 3-step library screen. (B) Amount of secreted laminin-α2 was measured by ELISA; cell growth was monitored by WTS8 assay. Groups were analysed by one-way ANOVA and compared to reference by Dunnett’s test in (B). **p<0.01, ***p<0.001, ****p<0.0001. **Figure S17: Specificity check of antibodies used in immunostaining.** Healthy skin cryosections were stained for the indicated antigens. Signals surrounding nuclear stains or colocalized to CD68 signals respectively indicated cellular or macrophage specificity. HF, hair follicle. Scale, 50um. (PDF 33538 KB)

## Data Availability

Sequencing data are available at GEO under accession numbers GSE223845 (sc-RNA-seq), GSE224703 and GSE224704 (bulk-RNA-seq, eosinophils and macrophages respectively). Processed sc-RNA-seq data used in meta-analysis (Fig. S13) are available at GEO accessions described in Table S1. All other data are included in the manuscript. Further data are available upon reasonable request to correspondence.

## Code availability

Generated codes are available upon reasonable request to correspondence.
