# Supplementary material for: Guided monocyte fate to FRβ/CD163+ S1 macrophage antagonises atopic dermatitis via fibroblastic matrices in mouse hypodermis
Source: Cell Mol Life Sci. 2024 Dec 25;82(1):14. doi: 10.1007/s00018-024-05543-2 (PMC11669644; doi:10.1007/s00018-024-05543-2)
Supplement: Supplementary file 1 — Supplementary file1 (DOCX 17 KB) [file 18_2024_5543_MOESM1_ESM.docx]

**Table S1**

| Tissue, accession, isolated MOMF number n | Perturbation, duration, sampling | Source cell sorting method | S1 character correlation coefficient *Folr2*; *Cd163*; summed *Folr2/Cd163*; | % expressed by MOMF scoring top 10% S1 score (summed *Folr2/Cd163* counts>1) |
| --- | --- | --- | --- | --- |
| Back skin  GSE223845 n = 5281 | MC903-induced atopic dermatitis d0-d14 d0, d8, d15 | Bead enriched CD45+ | 0.815; 0.572; 0.815; | 100% |
| Colon muscularis  GSE148794 n = 3921 | DSS-induced colitis d0-d6 d0, d3, d6, d9, d12, d15 | Total cells | 0.747; 0.652; 0.769; | 95.7% |
| Muscles GSE195507 n = 16283 | Ageing 23 months end-point | CD11b+ | 0.835; 0.681; 0.847; | 99.3% |
| Muscles GSE213925 n = 11131 | Duchenne muscular dystrophy genetic wt-NSG, mdx-NSG, mdxD2-NSG | Total cells | 0.739; 0.481; 0.740; | 99.5% |
| Brain GSE158269 n = 7403 | JHMV-induced acute encephalomyelitis d0 d0, d3, d7 (brain); d21 (spinal cord) | CD45+ | 0.107: -0.005; 0.082; | 4.73% |
| Kidney GSE200115 n = 79851 | Bilateral ischemic reperfusion injury d0, 19min 0h=qui, 12h=d0, d1, d6, d28 | F4/80^hi^ CD11b^int^ | 0.140; 0.129; 0.163; | 12.8% |
| Kidney GSE180420 n = 11781 | Bilateral ischemic reperfusion injury d0, 23min or 30min d0, d1, d3, d14 | Total cells | 0.267; 0.240; 0.328; | 25.1% |
| Pleural cavity GSE189031 n = 26245 | *L. sigmodontis* infection d0 d0, d35 | CD19-Ly6G-Nk1.1-CD90-CD5-IgM-CD3e-SiglecF-Ter119-CD45+CD11b+ | 0.295; 0.060; 0.236; | 46.6% |
| Adipose tissues  GSE161872 n = 4858 | High-fat diet 2 months end-point | Total cells | 0.763; 0.495; 0.776; | 99.8% |
| Lung GSE201698 n = 3445 | Bleomycin-induced fibrosis d0 d0, d3, d7, d10, d14, d21 | Total cells | 0.575; 0.435; 0.571; | 52.3% |
| Lung GSE202001 n = 53407 | Influenza A viral infection  d0 d0, d7, d14, d30 | CD45+CD64+SiglecF^hi/lo^ CD11b^lo/+^ and CD45+CD64+CD11b+MHCII- pooled | 0.294; 0.133; 0.293; | 19.0% |
| Small intestine muscularis GSE167460 n = 4792 | operative manipulation d0 d0, d1, d3 | CD45+ | 0.461; 0.291; 0.503; | 80.4% |
| Breast tumor GSE150675 n = 7242 | Localized ablative immunotherapy d0 d0, d9 | Bead depleted EpCAM-, CD45+ | 0.074; -0.003; 0.072; | 5.94% |

**Table S1: Datasets of various pathophysiological conditions used for meta-analysis and S1 marking efficiencies of *Cd163*/*Folr2*.** Macrophages were isolated from datasets achieved on Gene Expression Omnibus database and elemental subtype characters were computed based on gene signature described in Fig.1C. Marking efficiency (correlation and expression percentage) of Cd163/Folr2 transcripts for S1 character were examined.
