# Supplementary material for: Guided monocyte fate to FRβ/CD163+ S1 macrophage antagonises atopic dermatitis via fibroblastic matrices in mouse hypodermis
Source: Cell Mol Life Sci. 2024 Dec 25;82(1):14. doi: 10.1007/s00018-024-05543-2 (PMC11669644; doi:10.1007/s00018-024-05543-2)
Supplement: Supplementary file 4 — Supplementary Figure S1: Dynamics of macrophages and other leukocytes during atopic dermatitis. (A) Leukocyte composition in AD progression was shown. Monocyte/macrophages were isolated from the sc transcriptomes for downstream analyses. (B) Inflamed area were monitored following AD induction with MC903 on back skin of PDGFRα-H2BGFP knock-in mice. n = 14 mice for d0, d2 and d4 or 17 mice for d7. (C) Fold ratio of indicated cells to PDGFRα+ stromal cells in healthy/AD skin regions paired within the same mouse was measured by flow cytometry. n = 6 mice for mast and basophil; n = 7 mice for leukocyte, eosinophil and endothelial cell. Data described in Fig. 1D-F were analysed for (D) expression specificity on MOMF, eosinophil and stromal cells or (E) marking specificity of CD163 and FRβ for F4/80+ macrophages in healthy or AD skin. Groups were compared by paired t-test or Wilcoxon signed-sum test in (C). *p<0.05, **p<0.01, ***p<0.001. Figure S2: Gating strategy for identification of stromal cells, granulocytes and macrophage subtypes. Identification of (A) mast cells, basophils and (B) eosinophils for fold ratio quantification to PDGFRα+ stromal cells in Fig. S1C were shown. In (B-C), identification of macrophage subtypes quantified in Fig. 1D-F were shown as (I-VI). Figure S3: Expression of CD163 and FRβ in time course during PB monocyte differentiation. (A) Gene expression of CSFs in skin were examined in the indicated cell types with sc transcriptomes. (B) PB monocytes were differentiated with 20ng/ml M-CSF for the indicated period followed by flow cytometry measurement of CD163 and FRβ expression on F4/80+CD64+ macrophages. n = 4, 8 and 6 batches for d4, d6 and d8. (C) Differentiating PB monocytes were cocultured with the indicated cell mixture as described in Fig. 2B for 6 days. n = 6 batches for ctrl and complete coculture (all), and n = 4 for the rest. Each batch is derived from an individual mouse; sample marked with (ref) served as batch-specific internal ctrl. [file 18_2024_5543_MOESM4_ESM.pdf]

Fig.S1

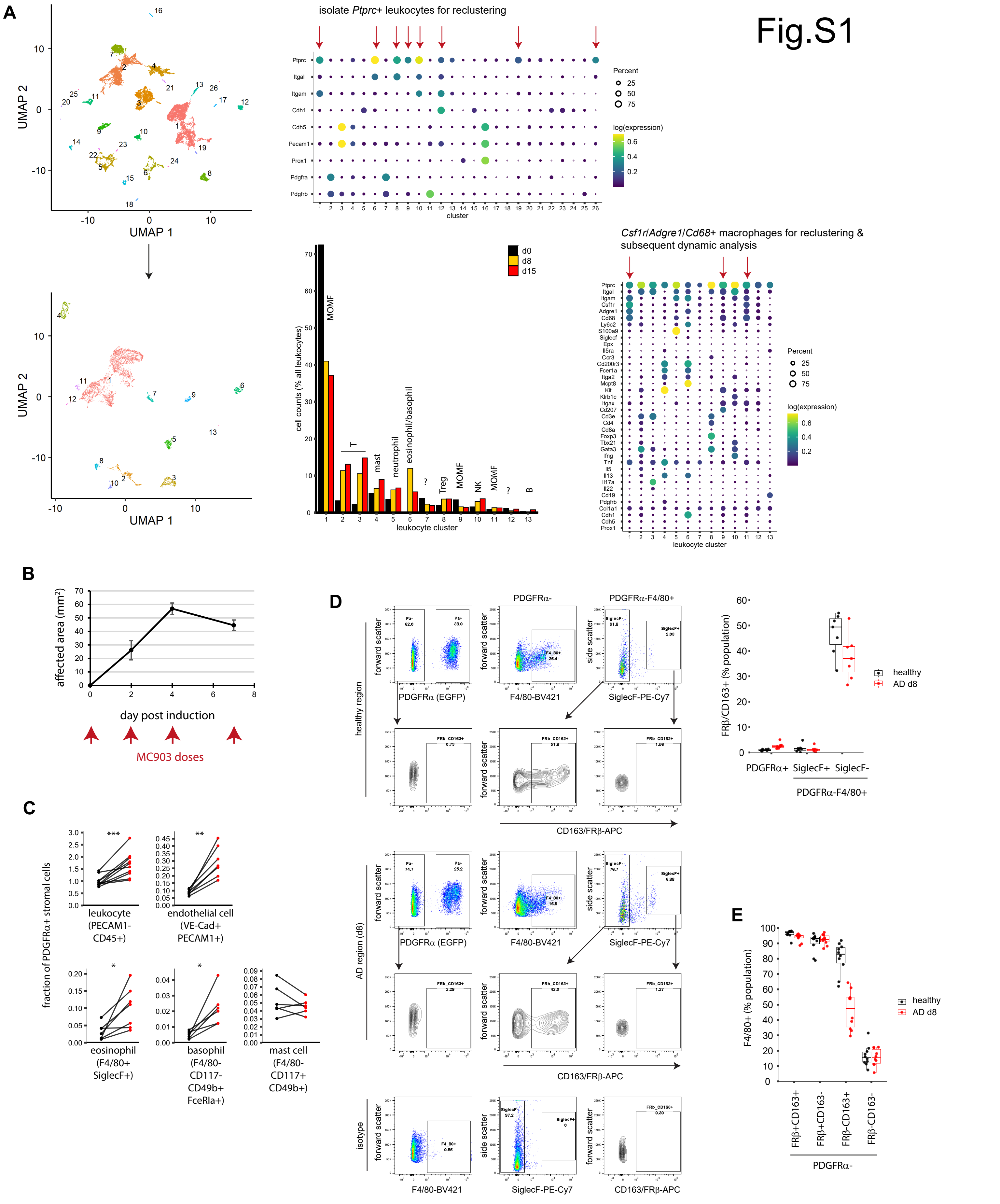

A
isolate Ptprc+ leukocytes for reclustering
UMAP 1
UMAP 2
Percent
log(expression)
cluster
cell counts (% all leukocytes)
leukocyte cluster
d0
d8
d15
MOMF
T
mast
neutrophil
eosinophil/basophil
Treg
NK
B
Csf1r/Adgre1/Cd68+ macrophages for reclustering & subsequent dynamic analysis
B
affected area (mm²)
day post induction
MC903 doses
C
fraction of PDGFRα+ stromal cells
leukocyte (PECAM1- CD45+)
endothelial cell (VE-Cad+ PECAM1+)
eosinophil (F4/80+ SiglecF+)
basophil (F4/80- CD117- CD49b+ FceRIa+)
mast cell (F4/80- CD117+ CD49b+)
D
healthy region
AD region (d8)
isotype
PDGFRα-
PDGFRα-F4/80+
forward scatter
side scatter
PDGFRα (EGFP)
F4/80-BV421
SiglecF-PE-Cy7
CD163/FRβ-APC
FRβ/CD163+ (% population)
healthy
AD d8
PDGFRα+
SiglecF+
SiglecF-
E
F4/80+ (% population)
FRβ+CD163+
FRβ+CD163-
FRβ-CD163+
FRβ-CD163-

Fig.S2

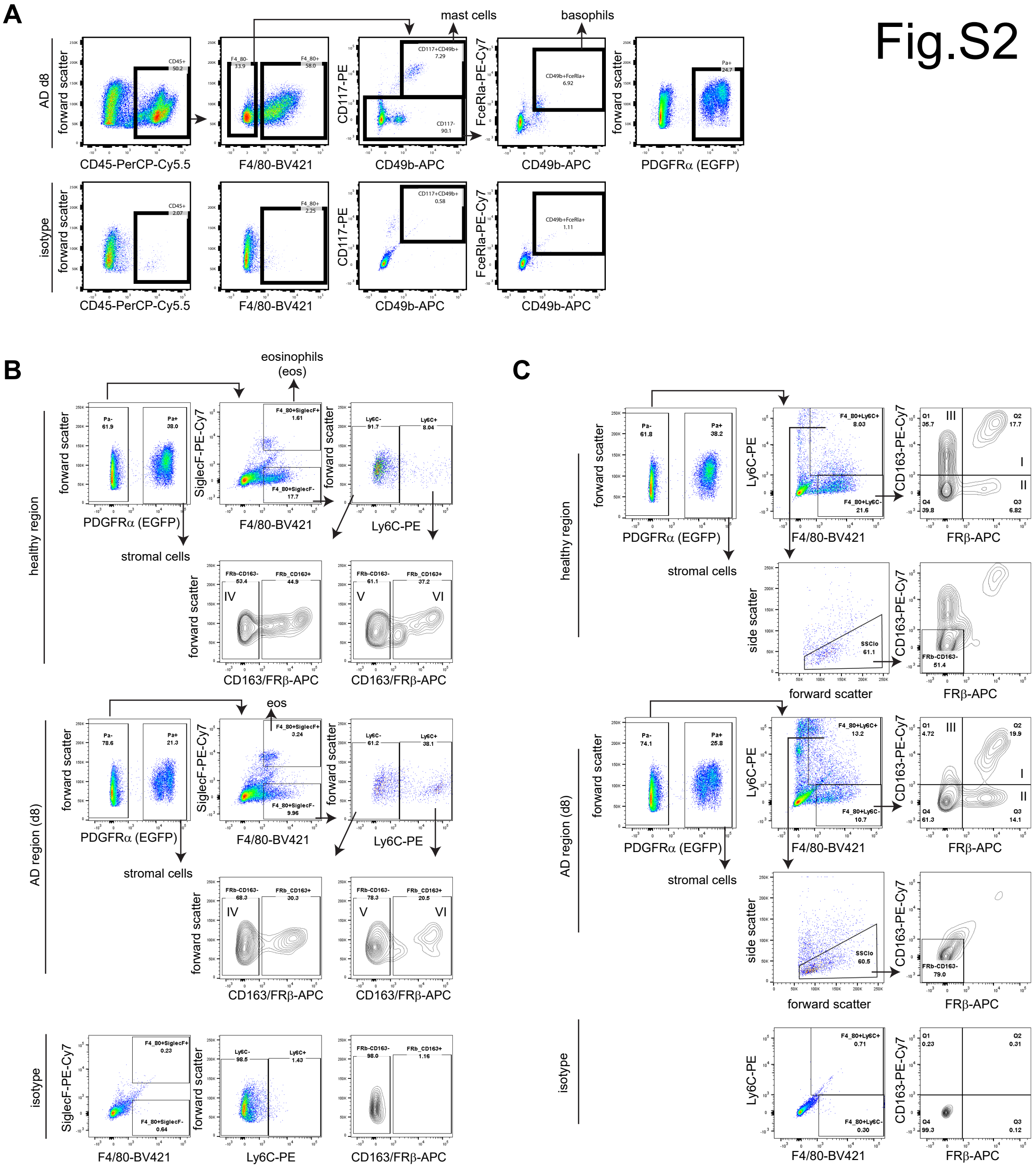
A
AD d8
isotype
forward scatter
CD45-PerCP-Cy5.5
F4/80-BV421
CD117-PE
CD49b-APC
FceRIa-PE-Cy7
PDGFRα (EGFP)
mast cells
basophils
B
C
healthy region
AD region (d8)
isotype
eosinophils (eos)
eos
stromal cells
SiglecF-PE-Cy7
Ly6C-PE
CD163/FRβ-APC
CD163-PE-Cy7
FRβ-APC
side scatter
forward scatter
I
II
III
IV
V
VI

Fig.S3

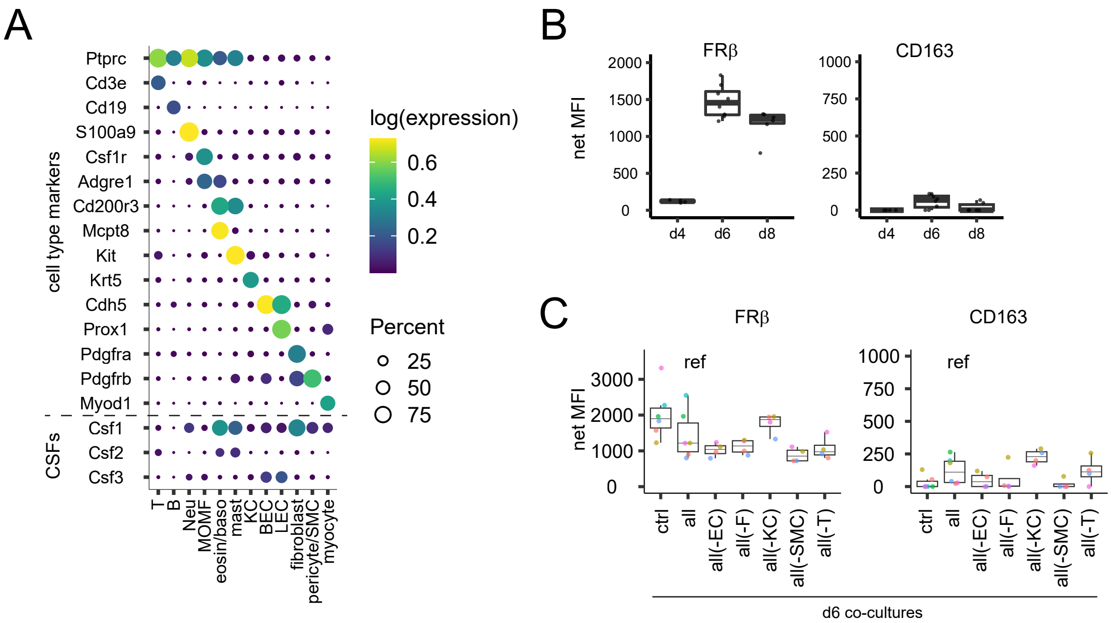
A
Ptprc
Cd3e
Cd19
S100a9
Csf1r
Adgre1
Cd200r3
Mcpt8
Kit
Krt5
Cdh5
Prox1
Pdgfra
Pdgfrb
Myod1
Csf1
Csf2
Csf3
cell type markers
CSFs
T
B
Neu
MOMF
eosin/baso
mast
KC
BEC
LEC
fibroblast
pericyte/SMC
myocyte
log(expression)
0.6
0.4
0.2
Percent
25
50
75
B
FRβ
CD163
net MFI
2000
1500
1000
500
0
750
250
d4
d6
d8
C
FRβ
CD163
ref
net MFI
3000
2000
1000
0
1000
750
500
250
ctrl
all
all(-EC)
all(-F)
all(-KC)
all(-SMC)
all(-T)
d6 co-cultures

Fig.S4

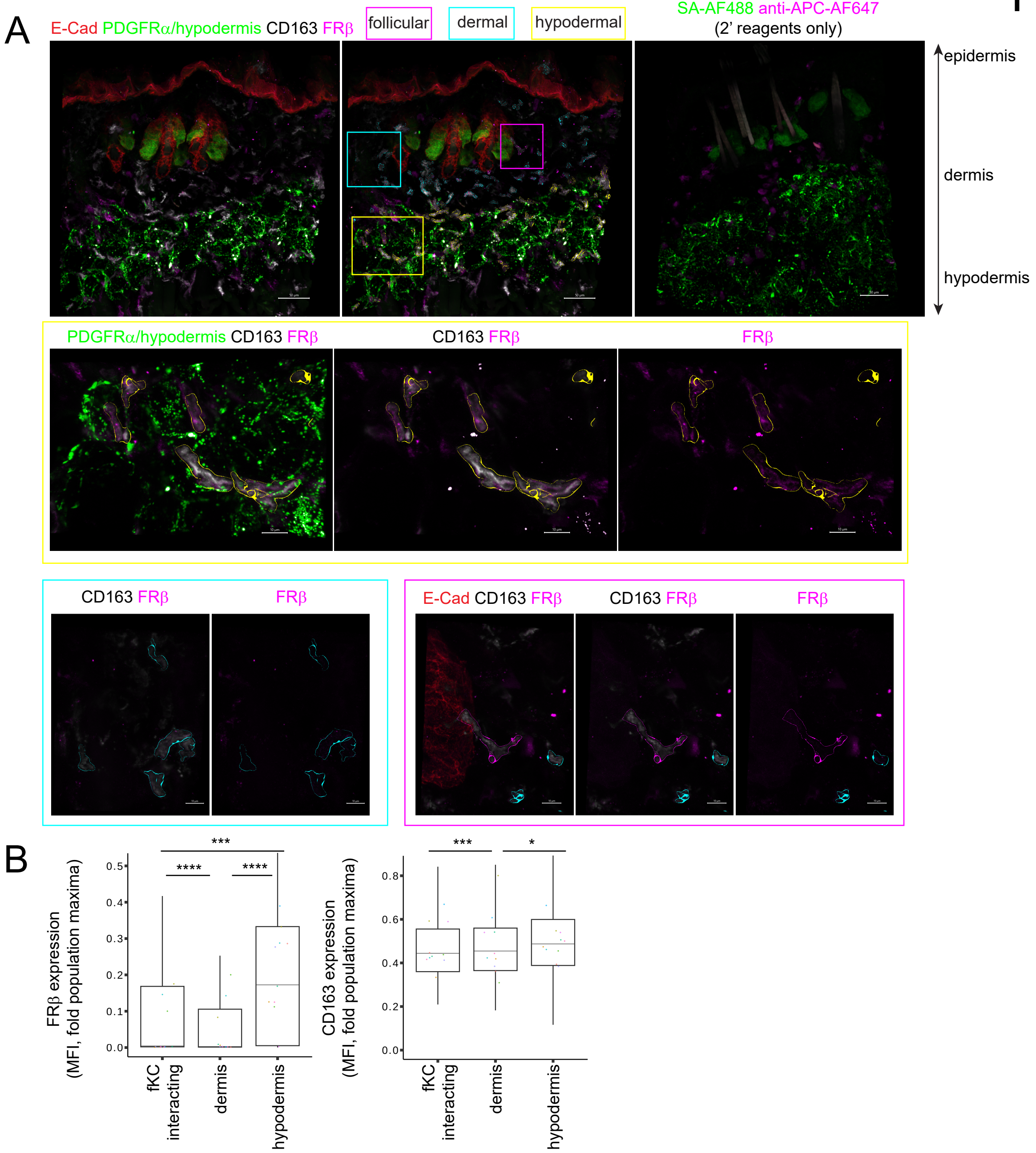

A
E-Cad PDGFRα/hypodermis CD163 FRβ
follicular
dermal
hypodermal
SA-AF488 anti-APC-AF647
(2' reagents only)
epidermis
dermis
hypodermis
PDGFRα/hypodermis CD163 FRβ
CD163 FRβ
FRβ
CD163 FRβ
FRβ
E-Cad CD163 FRβ
CD163 FRβ
FRβ
B
FRβ expression
(MFI, fold population maxima)
0.5
0.4
0.3
0.2
0.1
0.0
***
****
****
fKC
interacting
dermis
hypodermis
CD163 expression
(MFI, fold population maxima)
0.8
0.6
0.4
0.2
0.0
***
*
fKC
interacting
dermis
hypodermis

Fig.S5

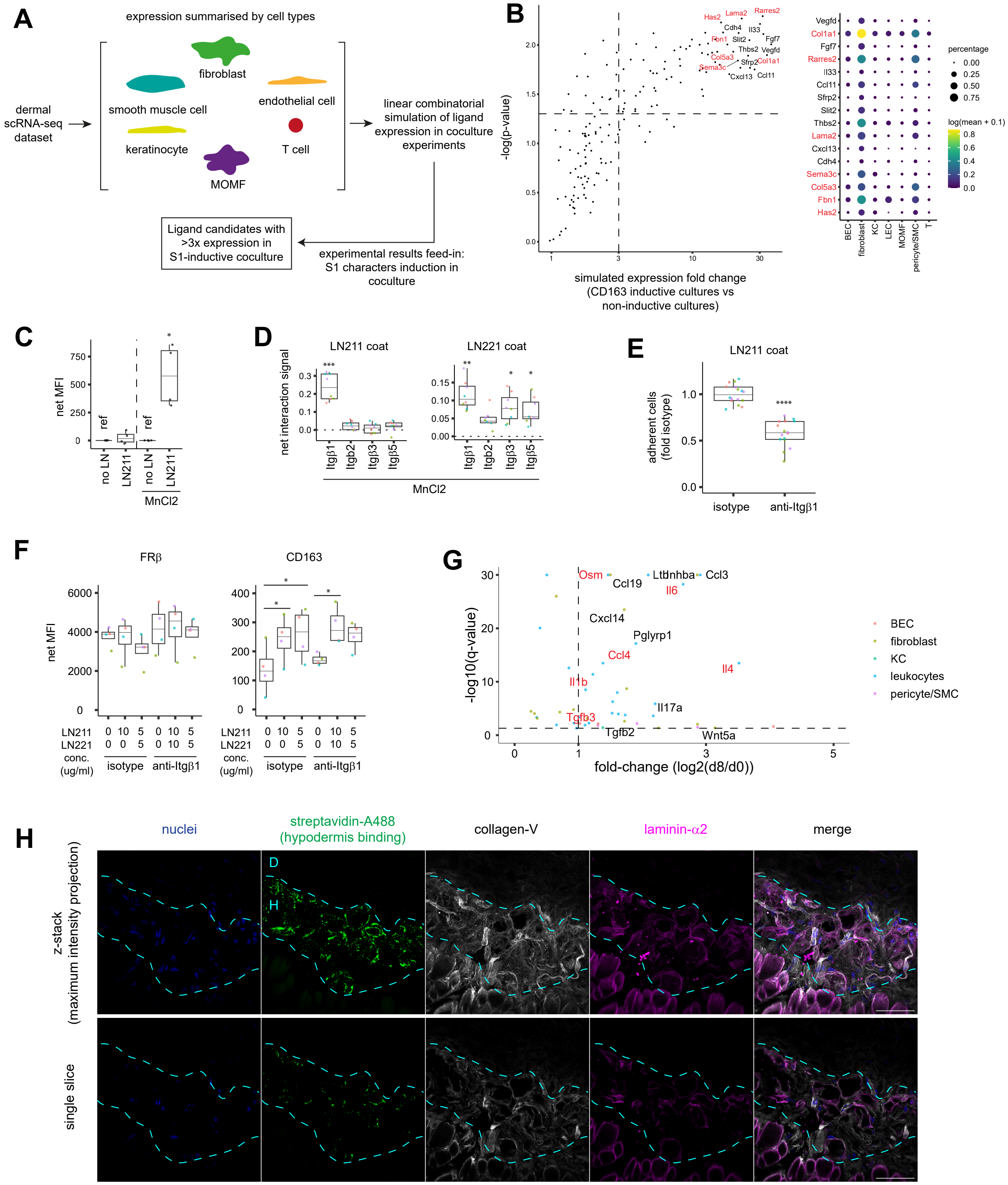

A
expression summarised by cell types
dermal scRNA-seq dataset
fibroblast
smooth muscle cell
endothelial cell
keratinocyte
T cell
MOMF
linear combinatorial simulation of ligand expression in coculture experiments
Ligand candidates with >3x expression in S1-inductive coculture
experimental results feed-in: S1 characters induction in coculture
B
-log(p-value)
simulated expression fold change (CD163 inductive cultures vs non-inductive cultures)
Has2
Lama2
Rarres2
Cdh4
Il33
Fbn1
Slit2
Fgf7
Thbs2
Vegfd
Col5a3
Sfrp2
Col1a1
Sema3c
Cxcl13
Ccl11
percentage
log(mean + 0.1)
BEC
fibroblast
KC
LEC
MOMF
pericyte/SMC
T
C
net MFI
ref
no LN
LN211
MnCl2
D
LN211 coat
LN221 coat
net interaction signal
Itgβ1
Itgb2
Itgβ3
Itgβ5
MnCl2
E
LN211 coat
adherent cells (fold isotype)
isotype
anti-Itgβ1
F
FRβ
CD163
net MFI
LN211
LN221
conc. (ug/ml)
isotype
anti-Itgβ1
G
-log10(q-value)
fold-change (log2(d8/d0))
Osm
Ccl19
Ltb
Inhba
Ccl3
Il6
Cxcl14
Pglyrp1
Ccl4
Il4
Il1b
Il17a
Tgfb3
Tgfb2
Wnt5a
BEC
fibroblast
KC
leukocytes
pericyte/SMC
H
nuclei
streptavidin-A488 (hypodermis binding)
collagen-V
laminin-α2
merge
z-stack (maximum intensity projection)
single slice
D
H

Fig.S6

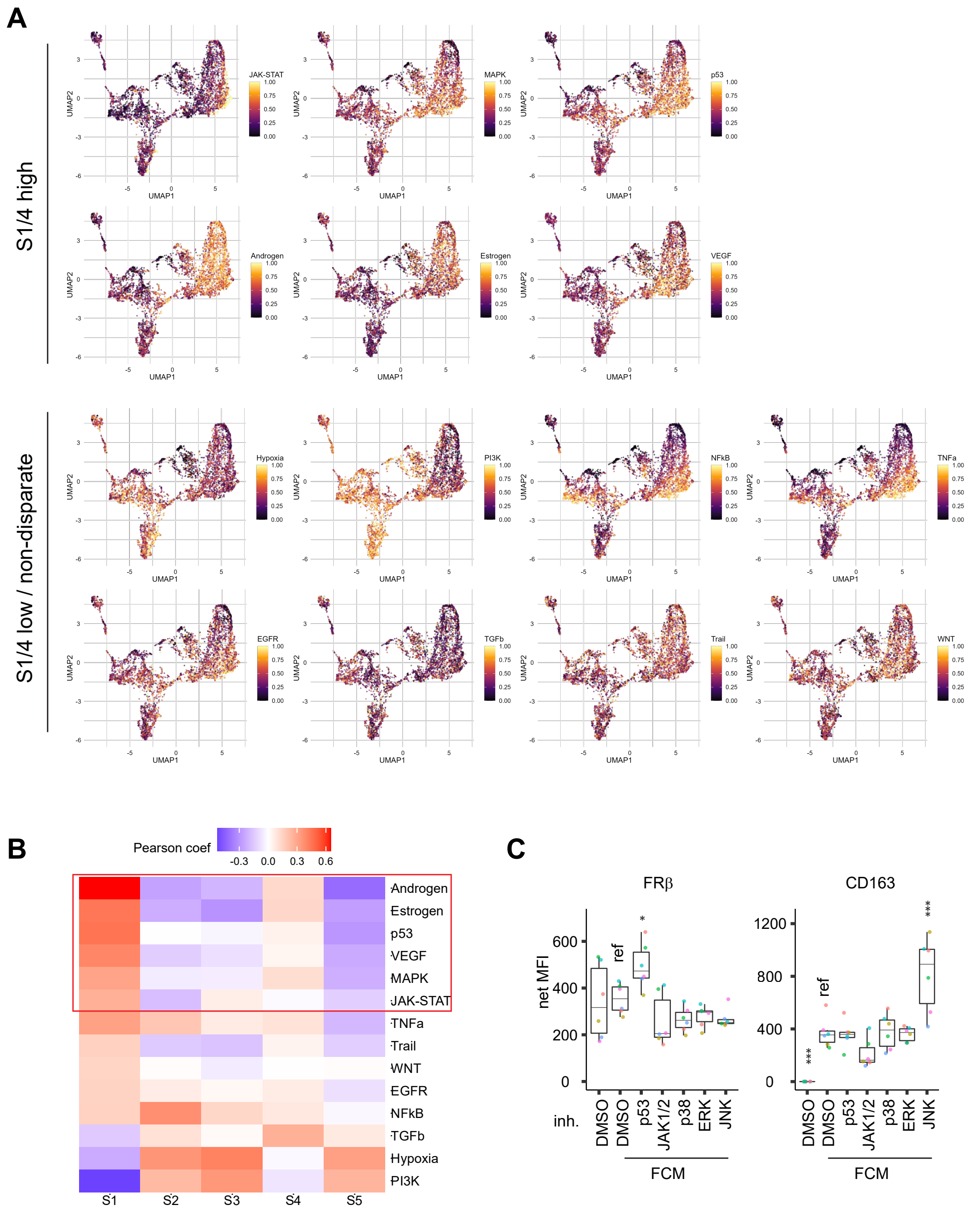
A
S1/4 high
S1/4 low / non-disparate
JAK-STAT
MAPK
p53
Androgen
Estrogen
VEGF
Hypoxia
PI3K
NFkB
TNFa
EGFR
TGFb
Trail
WNT
UMAP1
UMAP2
B
Pearson coef
-0.3
0.0
0.3
0.6
Androgen
Estrogen
p53
VEGF
MAPK
JAK-STAT
TNFa
Trail
WNT
EGFR
NFkB
TGFb
Hypoxia
PI3K
S1
S2
S3
S4
S5
C
FRβ
CD163
net MFI
600
400
200
0
1200
800
ref
inh.
DMSO
DMSO
p53
JAK1/2
p38
ERK
JNK
FCM

Fig.S7

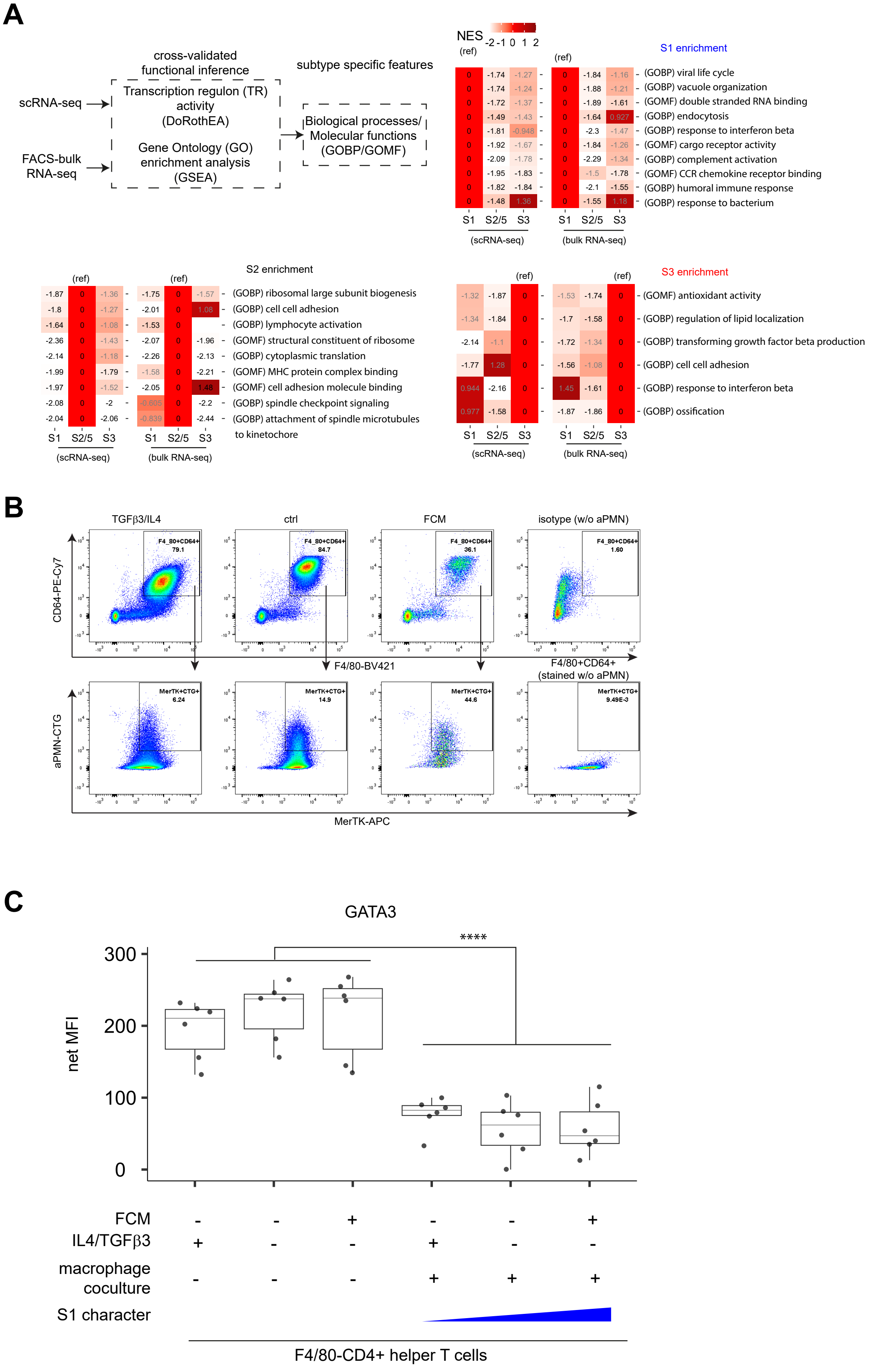

A
cross-validated functional inference
scRNA-seq
FACS-bulk RNA-seq
Transcription regulon (TR) activity (DoRothEA)
Gene Ontology (GO) enrichment analysis (GSEA)
subtype specific features
Biological processes/ Molecular functions (GOBP/GOMF)
NES
-2 -1 0 1 2
(ref)
S1 enrichment
(GOBP) viral life cycle
(GOBP) vacuole organization
(GOMF) double stranded RNA binding
(GOBP) endocytosis
(GOBP) response to interferon beta
(GOMF) cargo receptor activity
(GOBP) complement activation
(GOMF) CCR chemokine receptor binding
(GOBP) humoral immune response
(GOBP) response to bacterium
S1 S2/5 S3
(scRNA-seq)
(bulk RNA-seq)
S2 enrichment
(GOBP) ribosomal large subunit biogenesis
(GOBP) cell cell adhesion
(GOBP) lymphocyte activation
(GOMF) structural constituent of ribosome
(GOBP) cytoplasmic translation
(GOMF) MHC protein complex binding
(GOMF) cell adhesion molecule binding
(GOBP) spindle checkpoint signaling
(GOBP) attachment of spindle microtubules to kinetochore
S3 enrichment
(GOMF) antioxidant activity
(GOBP) regulation of lipid localization
(GOBP) transforming growth factor beta production
(GOBP) cell cell adhesion
(GOBP) response to interferon beta
(GOBP) ossification
B
TGFβ3/IL4
ctrl
FCM
isotype (w/o aPMN)
CD64-PE-Cy7
F4_80+CD64+
79.1
84.7
36.1
1.60
F4/80-BV421
F4/80+CD64+ (stained w/o aPMN)
aPMN-CTG
MerTK+CTG+
6.24
14.9
44.6
9.49E-3
MerTK-APC
C
GATA3
****
net MFI
300
200
100
0
FCM
IL4/TGFβ3
macrophage coculture
S1 character
F4/80-CD4+ helper T cells

Fig.S8

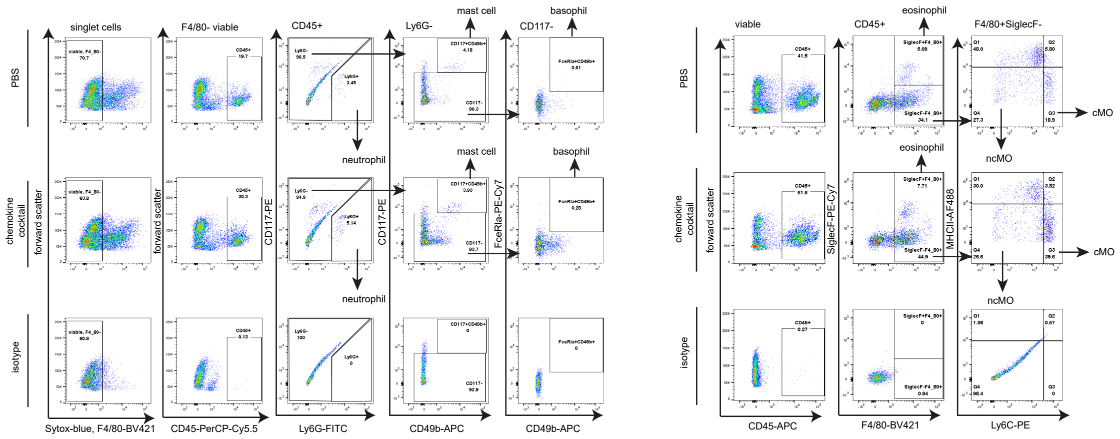

singlet cells
F4/80- viable
CD45+
Ly6G-
mast cell
CD117-
basophil
PBS
chemokine cocktail
isotype
forward scatter
CD117-PE
FceRIa-PE-Cy7
neutrophil
Sytox-blue, F4/80-BV421
CD45-PerCP-Cy5.5
Ly6G-FITC
CD49b-APC
viable
eosinophil
F4/80+SiglecF-
cMO
ncMO
SiglecF-PE-Cy7
MHCII-AF488
CD45-APC
F4/80-BV421
Ly6C-PE

Fig.S9

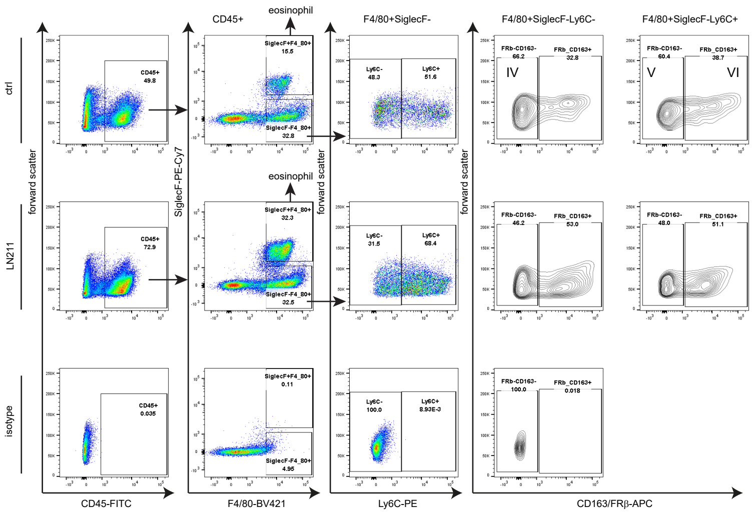

ctrl
LN211
isotype
forward scatter
CD45+
eosinophil
F4/80+SiglecF-
F4/80+SiglecF-Ly6C-
F4/80+SiglecF-Ly6C+
SiglecF-PE-Cy7
CD45-FITC
F4/80-BV421
Ly6C-PE
CD163/FRβ-APC
IV
V
VI

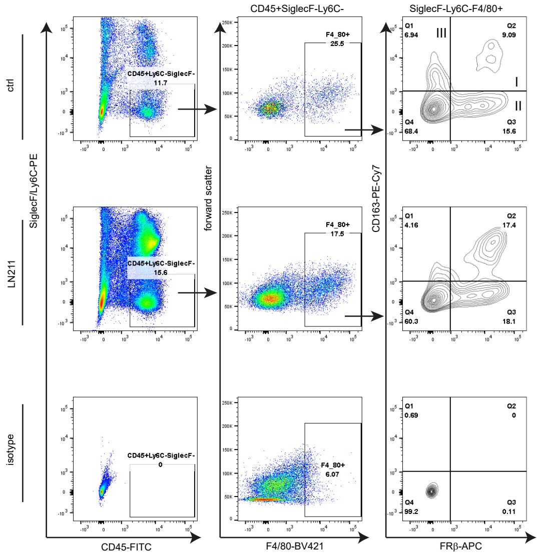

CD45+SiglecF-Ly6C-
SiglecF-Ly6C-F4/80+
ctrl
LN211
isotype
SiglecF/Ly6C-PE
forward scatter
CD163-PE-Cy7
CD45-FITC
F4/80-BV421
FRβ-APC
I
II
III

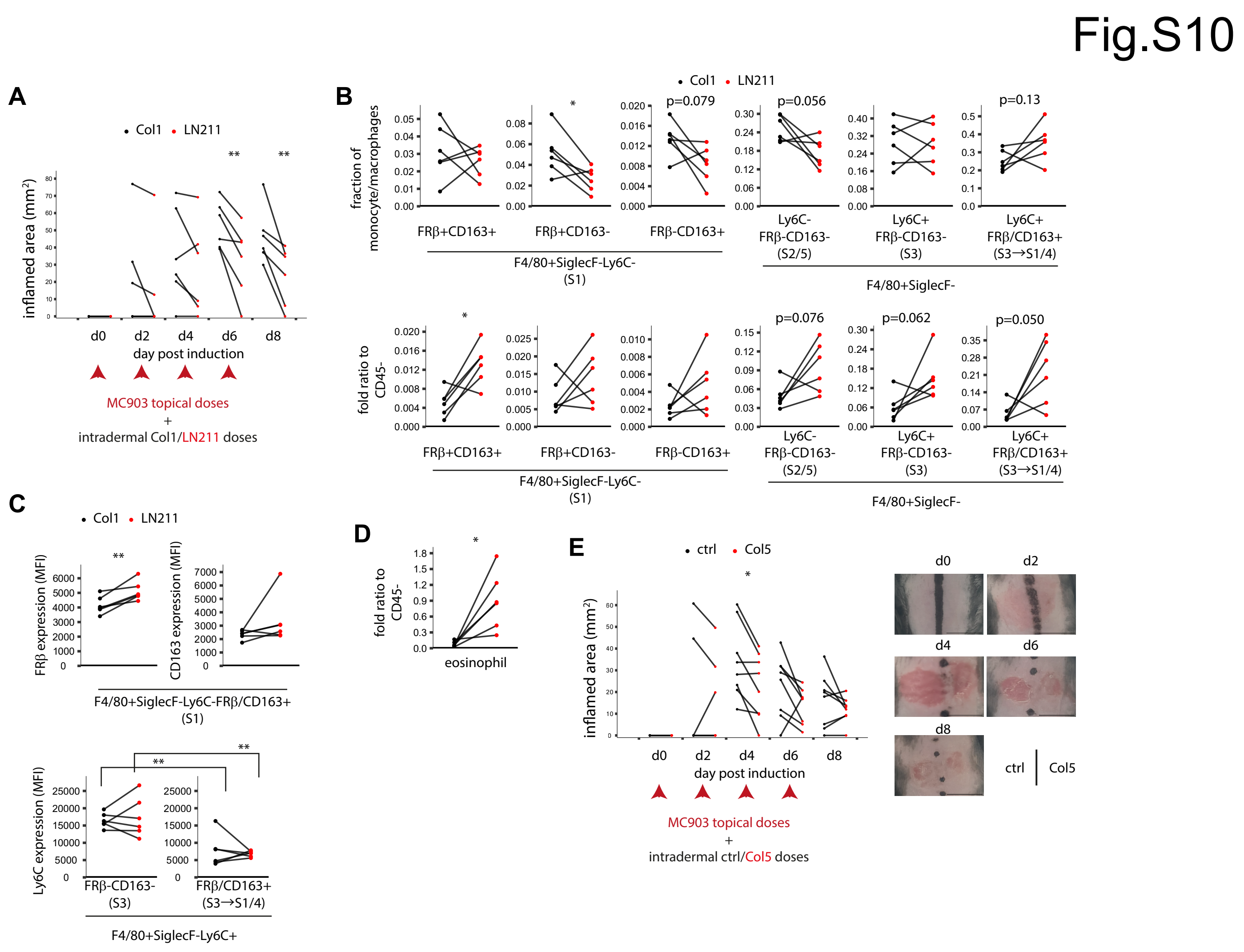

Fig.S10
A
Col1
LN211
inflamed area (mm²)
**
**
d0
d2
d4
d6
d8
day post induction
MC903 topical doses
+
intradermal Col1/LN211 doses
B
Col1
LN211
fraction of monocyte/macrophages
*
p=0.079
p=0.056
p=0.13
FRβ+CD163+
FRβ+CD163-
FRβ-CD163+
Ly6C- FRβ-CD163- (S2/5)
Ly6C+ FRβ-CD163- (S3)
Ly6C+ FRβ/CD163+ (S3→S1/4)
F4/80+SiglecF-Ly6C- (S1)
F4/80+SiglecF-
fold ratio to CD45-
*
p=0.076
p=0.062
p=0.050
C
Col1
LN211
FRβ expression (MFI)
CD163 expression (MFI)
**
F4/80+SiglecF-Ly6C-FRβ/CD163+ (S1)
Ly6C expression (MFI)
**
**
FRβ-CD163- (S3)
FRβ/CD163+ (S3→S1/4)
F4/80+SiglecF-Ly6C+
D
fold ratio to CD45-
*
eosinophil
E
ctrl
Col5
inflamed area (mm²)
*
d0
d2
d4
d6
d8
day post induction
MC903 topical doses
+
intradermal ctrl/Col5 doses
ctrl
Col5

Fig.S11

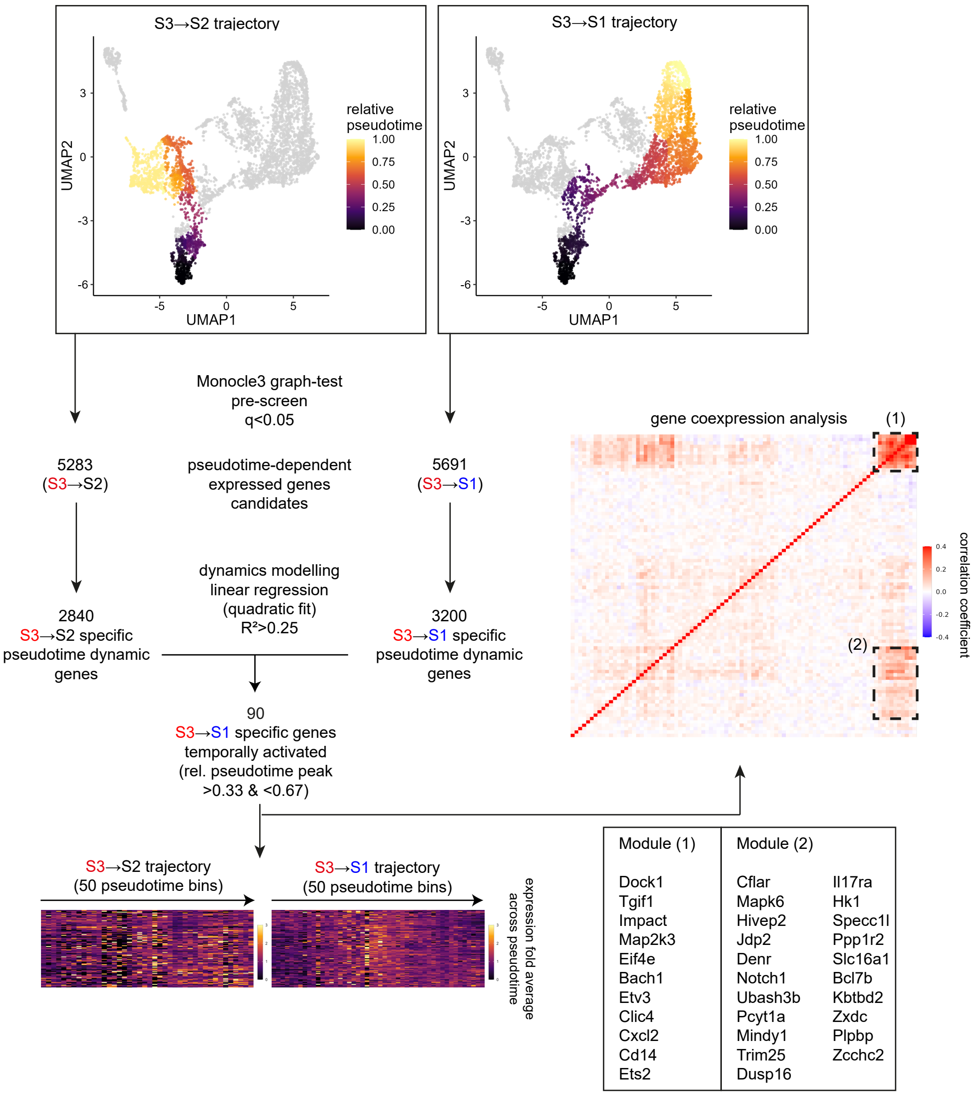

S3→S2 trajectory
S3→S1 trajectory
UMAP2
UMAP1
relative pseudotime
1.00
0.75
0.50
0.25
0.00
Monocle3 graph-test pre-screen q<0.05
5283 (S3→S2)
pseudotime-dependent expressed genes candidates
5691 (S3→S1)
dynamics modelling linear regression (quadratic fit) R²>0.25
2840 S3→S2 specific pseudotime dynamic genes
3200 S3→S1 specific pseudotime dynamic genes
90 S3→S1 specific genes temporally activated (rel. pseudotime peak >0.33 & <0.67)
gene coexpression analysis
(1)
(2)
correlation coefficient
S3→S2 trajectory (50 pseudotime bins)
S3→S1 trajectory (50 pseudotime bins)
expression fold average across pseudotime
Module (1)
Dock1
Tgif1
Impact
Map2k3
Eif4e
Bach1
Etv3
Clic4
Cxcl2
Cd14
Ets2
Module (2)
Cflar
Mapk6
Hivep2
Jdp2
Denr
Notch1
Ubash3b
Pcyt1a
Mindy1
Trim25
Dusp16
Il17ra
Hk1
Specc1l
Ppp1r2
Slc16a1
Bcl7b
Kbtbd2
Zxdc
Plpbp
Zcchc2

Fig.S12

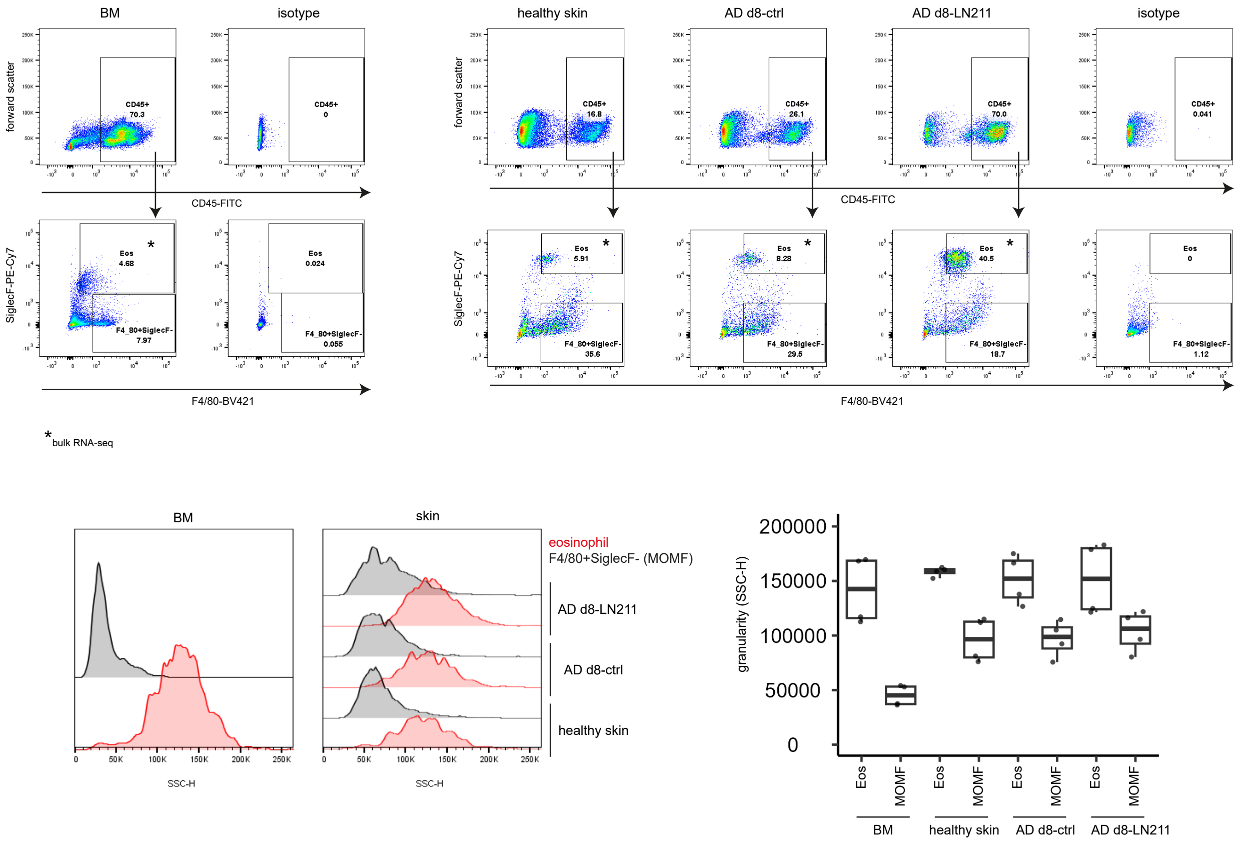
BM
isotype
healthy skin
AD d8-ctrl
AD d8-LN211
isotype
forward scatter
CD45+
70.3
CD45+
0
CD45+
16.8
CD45+
26.1
CD45+
70.0
CD45+
0.041
CD45-FITC
SiglecF-PE-Cy7
Eos
4.68
Eos
0.024
Eos
5.91
Eos
8.28
Eos
40.5
Eos
0
F4_80+SiglecF-
7.97
F4_80+SiglecF-
0.055
F4_80+SiglecF-
35.6
F4_80+SiglecF-
29.5
F4_80+SiglecF-
18.7
F4_80+SiglecF-
1.12
F4/80-BV421
* bulk RNA-seq
BM
skin
eosinophil
F4/80+SiglecF- (MOMF)
AD d8-LN211
AD d8-ctrl
healthy skin
SSC-H
granularity (SSC-H)
200000
150000
100000
50000
0
Eos
MOMF
BM
healthy skin
AD d8-ctrl
AD d8-LN211

Fig.S13

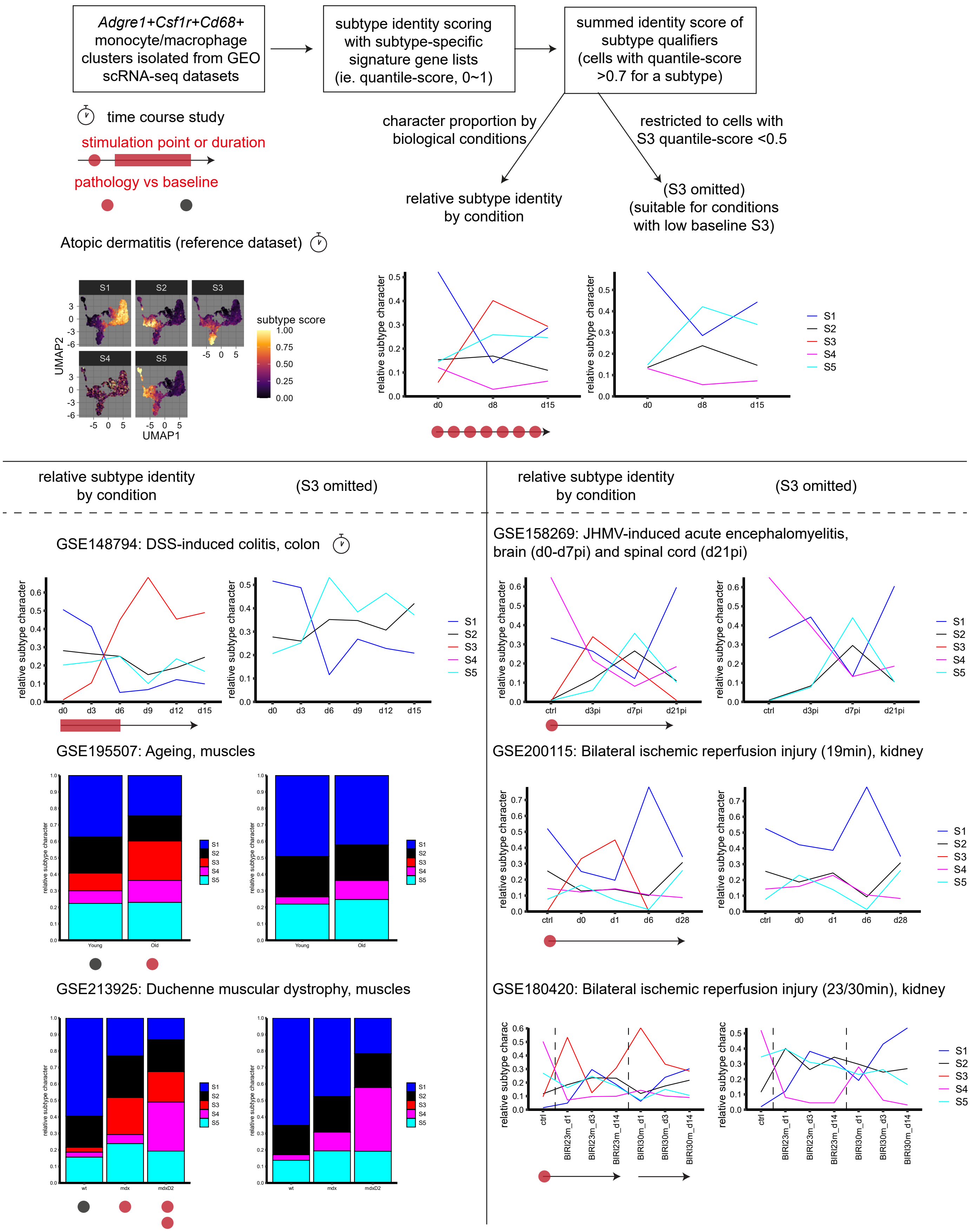

Adgre1+Csf1r+Cd68+ monocyte/macrophage clusters isolated from GEO scRNA-seq datasets
subtype identity scoring with subtype-specific signature gene lists (ie. quantile-score, 0~1)
summed identity score of subtype qualifiers (cells with quantile-score >0.7 for a subtype)
character proportion by biological conditions
restricted to cells with S3 quantile-score <0.5
relative subtype identity by condition
(S3 omitted) (suitable for conditions with low baseline S3)
time course study
stimulation point or duration
pathology vs baseline
Atopic dermatitis (reference dataset)
S1
S2
S3
S4
S5
subtype score
UMAP1
UMAP2
relative subtype character
d0
d8
d15
relative subtype identity by condition
(S3 omitted)
GSE148794: DSS-induced colitis, colon
d0
d3
d6
d9
d12
d15
GSE158269: JHMV-induced acute encephalomyelitis, brain (d0-d7pi) and spinal cord (d21pi)
ctrl
d3pi
d7pi
d21pi
GSE195507: Ageing, muscles
Young
Old
GSE200115: Bilateral ischemic reperfusion injury (19min), kidney
ctrl
d0
d1
d6
d28
GSE213925: Duchenne muscular dystrophy, muscles
wt
mdx
mdxD2
GSE180420: Bilateral ischemic reperfusion injury (23/30min), kidney
relative subtype charac
ctrl
BIRI23m_d1
BIRI23m_d3
BIRI23m_d14
BIRI30m_d1
BIRI30m_d3
BIRI30m_d14

Fig.S13 (continue)

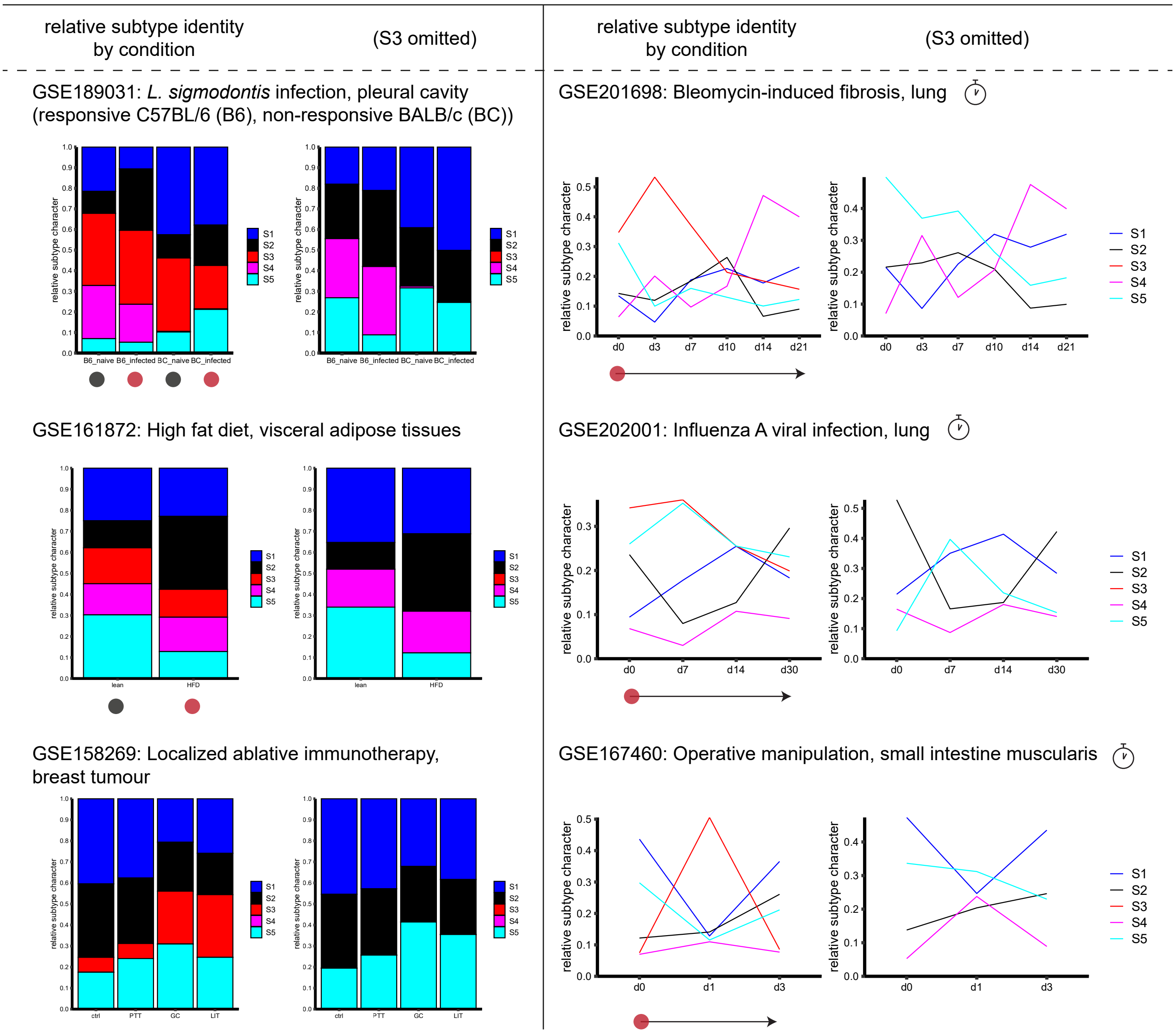

Fig.S14

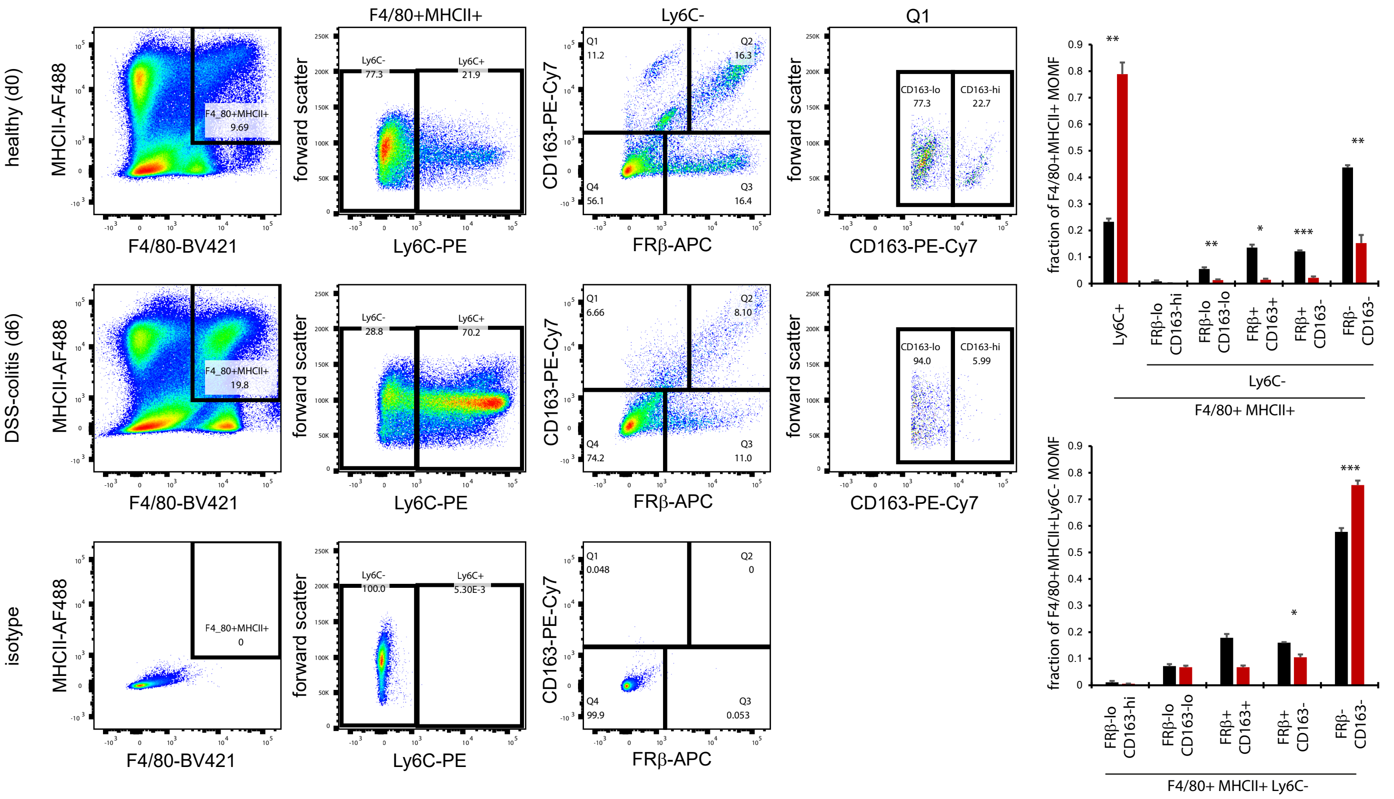

F4/80+MHCII+
Ly6C-
Q1
healthy (d0)
DSS-colitis (d6)
isotype
MHCII-AF488
F4/80-BV421
forward scatter
Ly6C-PE
CD163-PE-Cy7
FRβ-APC
F4_80+MHCII+
9.69
19.8
0
Ly6C-
77.3
Ly6C+
21.9
28.8
70.2
100.0
5.30E-3
Q1
11.2
Q2
16.3
Q4
56.1
Q3
16.4
6.66
8.10
74.2
11.0
0.048
0
99.9
0.053
CD163-lo
77.3
CD163-hi
22.7
94.0
5.99
fraction of F4/80+MHCII+ MOMF
Ly6C+
FRβ-lo CD163-hi
FRβ-lo CD163-lo
FRβ+ CD163+
FRβ+ CD163-
FRβ- CD163-
Ly6C-
F4/80+ MHCII+
fraction of F4/80+MHCII+Ly6C- MOMF
F4/80+ MHCII+ Ly6C-

Fig.S15

A

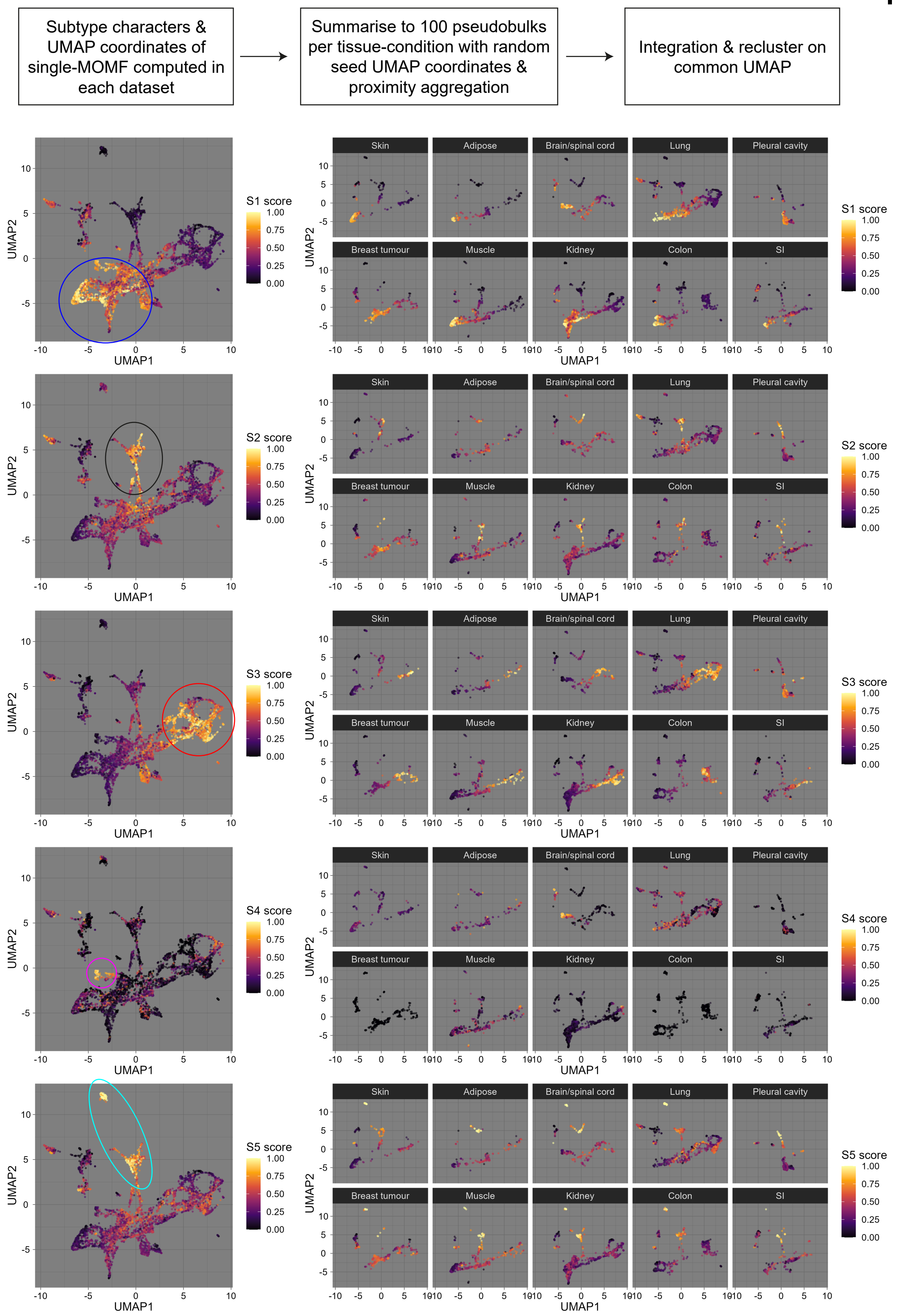

Subtype characters & UMAP coordinates of single-MOMF computed in each dataset
Summarise to 100 pseudobulks per tissue-condition with random seed UMAP coordinates & proximity aggregation
Integration & recluster on common UMAP
S1 score
S2 score
S3 score
S4 score
S5 score
1.00
0.75
0.50
0.25
0.00
UMAP1
UMAP2
Skin
Adipose
Brain/spinal cord
Lung
Pleural cavity
Breast tumour
Muscle
Kidney
Colon
SI

B

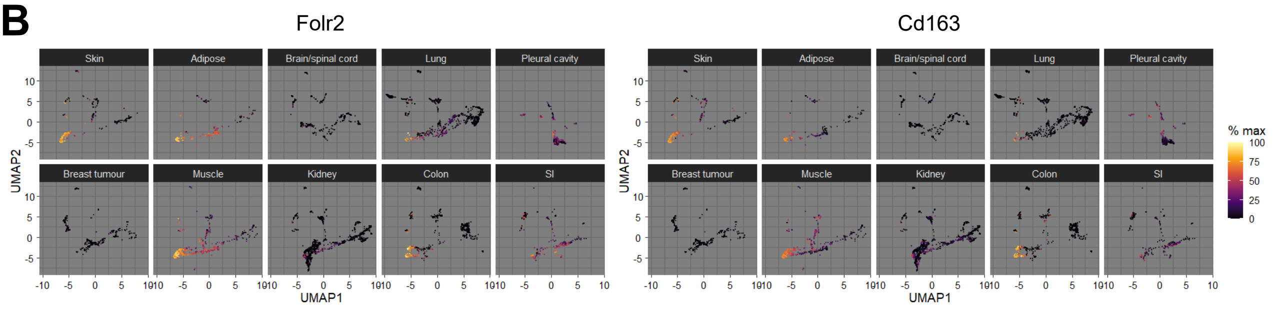

Folr2
Cd163
Skin
Adipose
Brain/spinal cord
Lung
Pleural cavity
Breast tumour
Muscle
Kidney
Colon
SI
% max
100
75
50
25
0
UMAP1
UMAP2

Fig.S16

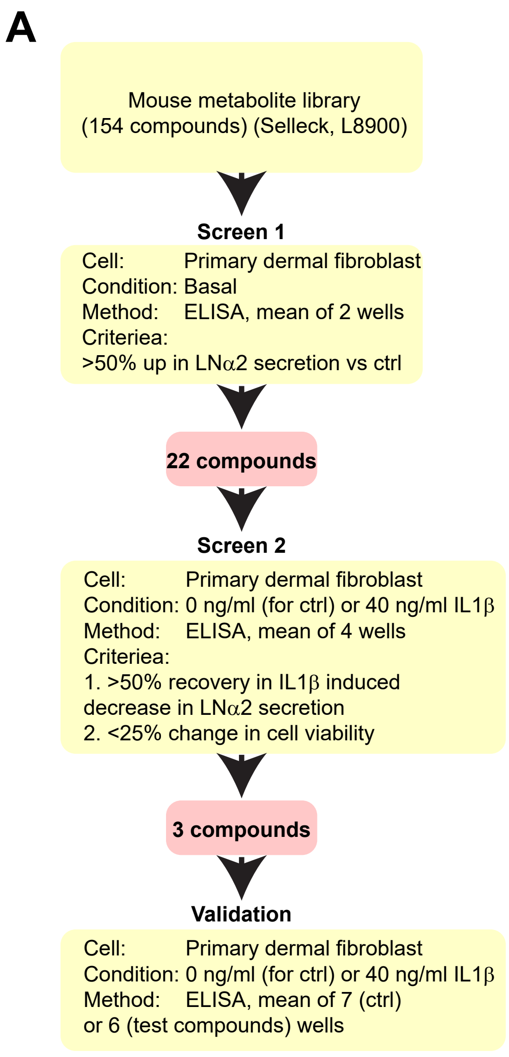

A
Mouse metabolite library
(154 compounds) (Selleck, L8900)
Screen 1
Cell: Primary dermal fibroblast
Condition: Basal
Method: ELISA, mean of 2 wells
Criteriea:
>50% up in LNα2 secretion vs ctrl
22 compounds
Screen 2
Cell: Primary dermal fibroblast
Condition: 0 ng/ml (for ctrl) or 40 ng/ml IL1β
Method: ELISA, mean of 4 wells
Criteriea:
1. >50% recovery in IL1β induced decrease in LNα2 secretion
2. <25% change in cell viability
3 compounds
Validation
Cell: Primary dermal fibroblast
Condition: 0 ng/ml (for ctrl) or 40 ng/ml IL1β
Method: ELISA, mean of 7 (ctrl) or 6 (test compounds) wells

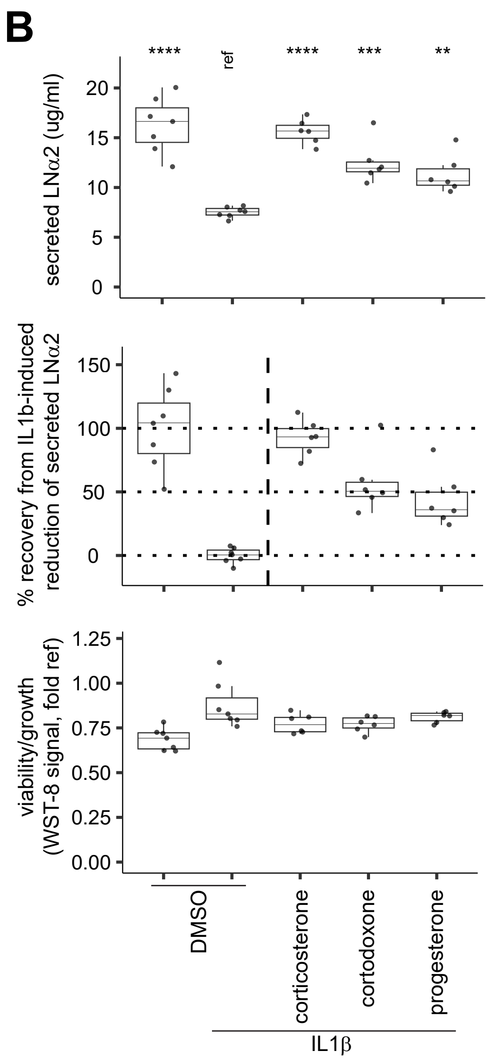

B
****
ref
****
***
**
secreted LNα2 (ug/ml)
0
5
10
15
20
% recovery from IL1b-induced reduction of secreted LNα2
0
50
100
150
viability/growth (WST-8 signal, fold ref)
0.00
0.25
0.50
0.75
1.00
1.25
DMSO
corticosterone
cortodoxone
progesterone
IL1β

Fig.S17

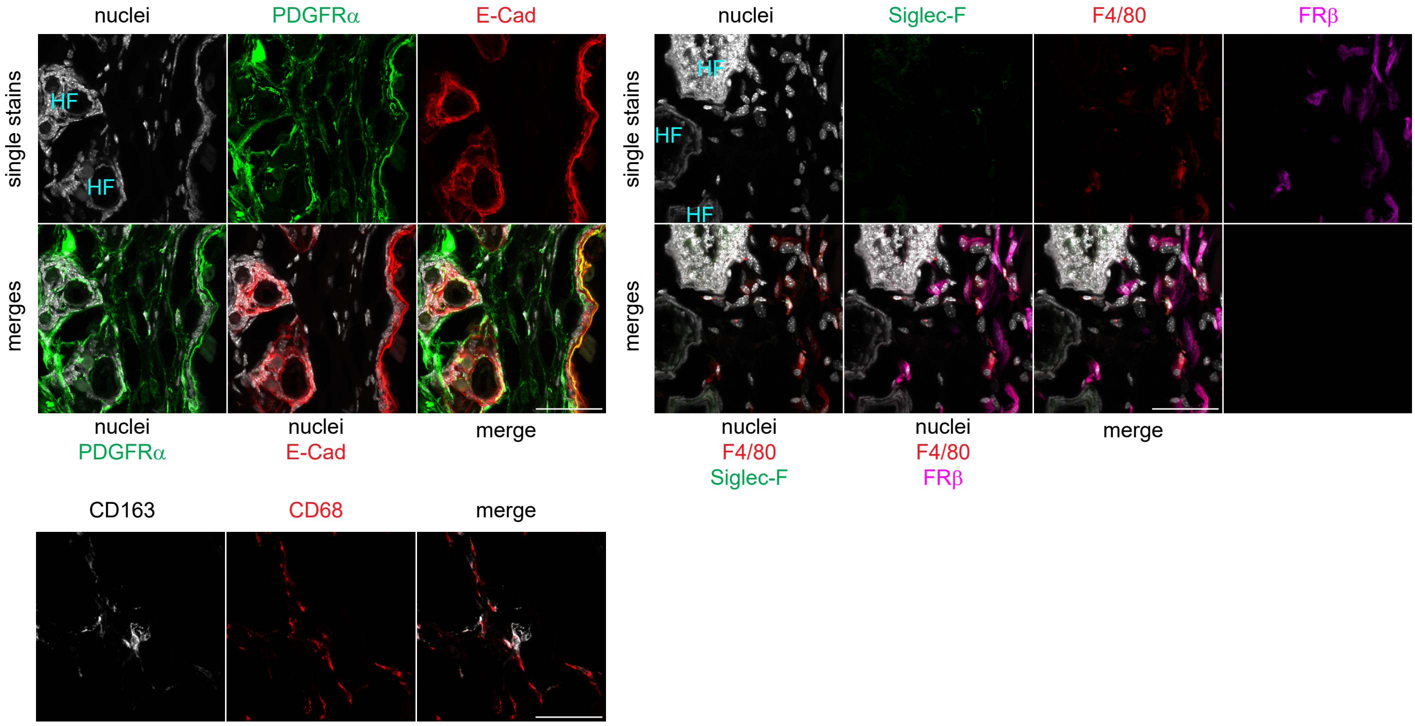
nuclei
PDGFRα
E-Cad
single stains
merges
HF
HF
nuclei PDGFRα
nuclei E-Cad
merge
nuclei
Siglec-F
F4/80
FRβ
single stains
merges
HF
HF
HF
nuclei F4/80 Siglec-F
nuclei F4/80 FRβ
merge
CD163
CD68
merge
